# Excess Pyrophosphate Restrains Pavement Cell Morphogenesis and Alters Organ Flatness in *Arabidopsis thaliana*

**DOI:** 10.3389/fpls.2020.00031

**Published:** 2020-02-21

**Authors:** Shizuka Gunji, Yoshihisa Oda, Hisako Takigawa-Imamura, Hirokazu Tsukaya, Ali Ferjani

**Affiliations:** ^1^ United Graduate School of Education, Tokyo Gakugei University, Tokyo, Japan; ^2^ Department of Gene Function and Phenomics, National Institute of Genetics, Mishima, Japan; ^3^ Department of Genetics, The Graduate University for Advanced Studies, SOKENDAI, Mishima, Japan; ^4^ Department of Anatomy and Cell Biology, Graduate School of Medical Sciences, Kyushu University, Fukuoka, Japan; ^5^ Department of Biological Sciences, Graduate School of Science, The University of Tokyo, Tokyo, Japan; ^6^ Department of Biology, Tokyo Gakugei University, Tokyo, Japan

**Keywords:** *Arabidopsis thaliana*, H^+^-PPase, *fugu5* mutant, PPi, pavement cells

## Abstract

In *Arabidopsis thaliana*, the vacuolar proton-pumping pyrophosphatase (H^+^-PPase) is highly expressed in young tissues, which consume large amounts of energy in the form of nucleoside triphosphates and produce pyrophosphate (PPi) as a byproduct. We reported that excess PPi in the H^+^-PPase loss-of-function *fugu5* mutant severely compromised gluconeogenesis from seed storage lipids, arrested cell division in cotyledonary palisade tissue, and triggered compensated cell enlargement; this phenotype was recovered upon sucrose supply. Thus, we provided evidence that the hydrolysis of inhibitory PPi, rather than vacuolar acidification, is the major contribution of H^+^-PPase during seedling establishment. Here, examination of the epidermis revealed that *fugu5* pavement cells exhibited defective puzzle-cell formation. Importantly, removal of PPi from *fugu5* background by the yeast cytosolic PPase IPP1, in *fugu5-1 AVP1_pro_::IPP1* transgenic lines, restored the phenotypic aberrations of *fugu5* pavement cells. Surprisingly, pavement cells in mutants with defects in gluconeogenesis (*pck1-2*) or the glyoxylate cycle (*icl-2*; *mls-2*) showed no phenotypic alteration, indicating that reduced sucrose production from seed storage lipids is not the cause of *fugu5* epidermal phenotype. *fugu5* had oblong cotyledons similar to those of *angustifolia-1* (*an-1*), whose leaf pavement cells display an abnormal arrangement of cortical microtubules (MTs). To gain insight into the genetic interaction between ANGUSTIFOLIA and H^+^-PPase in pavement cell differentiation, *an-1 fugu5-1* was analyzed. Surprisingly, epidermis developmental defects were synergistically enhanced in the double mutant. In fact, *an-1 fugu5-1* pavement cells showed a striking three-dimensional growth phenotype on both abaxial and adaxial sides of cotyledons, which was recovered by hydrolysis of PPi in *an-1 fugu5-1 AVP1_pro_::IPP1*. Live imaging revealed that cortical MTs exhibited a reduced velocity, were slightly fragmented and sparse in the above lines compared to the WT. Consistently, addition of PPi *in vitro* led to a dose-dependent delay of tubulin polymerization, thus supporting a link between PPi and MT dynamics. Moreover, mathematical simulation of three-dimensional growth based on cotyledon proximo-distal and medio-lateral phenotypic quantification implicated restricted cotyledon expansion along the medio-lateral axis in the crinkled surface of *an-1 fugu5-1*. Together, our data suggest that PPi homeostasis is a prerequisite for proper pavement cell morphogenesis, epidermal growth and development, and organ flattening.

## Introduction

Although housekeeping enzymes have been characterized in detail, molecular lesions in such genes are often associated with lethality, hampering assessment of the mechanism of their *in vivo* roles beyond housekeeping activities. For example, 3-phosphoglycerate dehydrogenase (PHGDH) catalyzes the first step of L-serine biosynthesis in animals (Klomp et al., 2000). PHGDH deficiency causes a disorder of L-serine biosynthesis that is characterized by congenital microcephaly, psychomotor retardation, and seizures (Klomp et al., 2000). Although L-serine is a non-essential amino acid, studies on patients with Neu-Laxova syndrome have suggested a fundamental role for PHGDH activity in metabolism, development, and function of the human central nervous system (Klomp et al., 2000). Moreover, several recent reports have indicated that some of these housekeeping enzymes perform a variety of other functions, such as acting as virulence factors for pathogens (Pancholi and Chhatwal, 2003; and references therein).

All above-ground organs of plants emerge at the shoot apical meristem (SAM). Plant leaves play a central role not only in capturing light for photosynthesis but also by sensing the environmental signals that are integrated to enable optimal growth. These functions are accomplished independently and cooperatively by the different cell types on the surface or embedded within plant leaf tissues. Leaf primordia evolve at the flanks of the SAM and undergo a phase of cell proliferation followed by cell differentiation (Donnelly et al., 1999; Ferjani et al., 2007). Proliferating cells are characterized by an active metabolism, whereby they consume large amounts of energy in the form of nucleoside triphosphates (NTPs). Simultaneously, in nearly 200 different metabolic reactions (Heinonen, 2001)—including DNA replication, amino acid activation, and protein and cell wall biosynthesis—they produce pyrophosphate (PPi). PPi is a toxic molecule that if not immediately hydrolyzed by soluble-type pyrophosphatases (sPPases) and/or membrane-bound pyrophosphatases (H^+^-PPases) (Ferjani et al., 2014; Segami et al., 2018), irreversibly arrests the above metabolic reaction.

Most studies of the physiological function(s) of PPi-hydrolyzing enzymes, which can be viewed as housekeeping enzymes, and the impact of excess PPi *in vivo*, have been unsuccessful (Ferjani et al., 2014). Indeed, the importance of PPi-homeostasis in development is evidenced by the many reports in several organisms. For example, decreased expression of *ppa*, encoding *E. coli* sPPase, raised the PPi level and arrested growth (Chen et al., 1990). Similarly, the cytosolic PPase IPP1 is essential for cell viability in *Saccharomyces cerevisiae* (Lundin et al., 1991). Moreover, the *Caenorhabditis elegans* sPPase *pyp-1* null mutant, displayed gross defects in intestinal morphology and function and was arrested at early larval stages (Ko et al., 2007).

The importance of PPi homeostasis in plant growth and development in *Arabidopsis thaliana* (Arabidopsis, hereafter) has been intensively investigated using *fugu5* mutants, harboring a molecular lesion in the vacuolar-type H^+^-PPase. For instance, we demonstrated that the H^+^-PPase is the major PPase in Arabidopsis (Ferjani et al., 2011; Ferjani et al., 2014; Asaoka et al., 2016; Segami et al., 2018). Failure to hydrolyze PPi led to developmental defects at the organism, organ, tissue, and cellular levels. Indeed, the *fugu5* mutant plants display retarded post-germinative growth and exhibit oblong-shaped cotyledons and compensation in their palisade tissue, such as excessive cell expansion triggered by decreased cell proliferation (Ferjani et al., 2007; Ferjani et al., 2008; Ferjani et al., 2011; Ferjani et al., 2012). In addition, gluconeogenesis, the process that produces sucrose (Suc) from triacylglycerol (TAG) in seed storage lipids, is partially suppressed in *fugu5* mutants (Ferjani et al., 2011; Takahashi et al., 2017).

Comparative metabolomics using capillary electrophoresis time-of-flight mass spectrometry (CE-TOF MS) combined with the mathematical theory structural sensitivity analysis unambiguously demonstrated that UDP-Glc pyrophosphorylase (UGPase) is the major target of the inhibitory effect of PPi *in vivo* (Ferjani et al., 2018). Moreover, stomatal closure delay and patterning defects in the *fugu5* background are specifically triggered by excess PPi within guard cells (Asaoka et al., 2019). Notably, specific removal of PPi by the expression of yeast sPPase IPP1 rescued all of the above developmental and metabolic defects. Therefore, most developmental defects triggered by excess PPi can be regarded as the result of metabolic defects, pointing to the importance of metabolic regulation in organogenesis. Despite being produced in all plant cells, the developmental and metabolic targets of PPi are unexpectedly specific, challenging the established concept of the role of housekeeping enzymes (Horiguchi et al., 2012; Tsukaya et al., 2013). Notably, the constitutive expression of H^+^-PPase promotes growth of plants confronted by a variety of abiotic stresses, rendering this enzyme of great interest to crop breeders (Wang et al., 2016; Schilling et al., 2017).

Epidermal pavement cells in Arabidopsis leaves grow into jigsaw shapes. However, why and how plants make such interlocking puzzle-shaped cells remains under investigation. Several proteins of the Rho guanidine triphosphatase (GTPase) of plants (ROP) family play a key role in shaping pavement cells. Pavement cell morphogenesis is regulated by the signaling of two ROP-mediated pathways with opposing effects on cell expansion (Fu et al., 2005). In fact, ROP2 activates RIC4 to promote lobe formation (actin) and ROP6 activates RIC1 to promote neck formation (MTs/cellulose) (Fu et al., 2005). Perturbation of the ROP/RIC pathways result in defective puzzle cell formation (Fu et al., 2002; Fu et al., 2005; Fu et al., 2009; Xu et al., 2010; Lin et al., 2013). The phytohormone auxin was proposed to act as an extracellular communication signal, provided that a lobe in one pavement cell is connected to the neck of its neighbor (Fu et al., 2005; Xu et al., 2010; Li et al., 2011; Lin et al., 2015), but this is still debated. The countersignaling of the above two pathways explains the interdigitating separation of the lobing and indenting domains of the cell cortex during the formation of interlocking puzzle-shaped cells. While the above studies unveiled key players in pavement cell patterning, the impact of metabolic regulation on this process is unclear.

Here, we propose PPi homeostasis as a key factor in puzzle cell patterning. We found that while excess PPi ions reduced the complexity of pavement cells, this recovered upon PPi hydrolysis but not Suc supply. Both *in vivo* live imaging of MTs and *in vitro* assays of tubulin polymerization indicated that the inhibitory effect of PPi within pavement cells could be in part explained by aberrant MT dynamics. Finally, excess PPi in the *angustifolia-1* (*an-1*) mutant background led to organs with wavy surfaces and three-dimensional (3D) protrusions, to which a computational model revealed that restriction of cotyledon expansion along the medio-lateral axis is a major contributor. Taken together, our findings suggest that excess PPi has different effects in different leaf tissues, namely the palisade and epidermis.

## Materials and Methods

### Plant Materials and Growth Conditions

The wild type (WT) used in this study was Columbia-0 (Col-0), and all other mutants and transgenic lines were in the Col-0 background. The three independent loss-of-function mutant alleles of H^+^-PPase used in this study, namely *fugu5-1*, *fugu5-2*, and *fugu5-3* have been all previously characterized (Ferjani et al., 2011; Fukuda et al., 2016). In addition, two previously described independent transgenic lines expressing yeast-soluble PPase IPP1 in the *fugu5-1* mutant background (*fugu5-1 AVP1_pro_::IPP1*#8-3 and *fugu5-1 AVP1_pro_::IPP1*#17-3) were used (Ferjani et al., 2011; Fukuda et al., 2016). Seeds of *icl-2*, *mls-2*, and *pck1-2* mutant lines, which have all been previously characterized in (Eastmond et al., 2000; Cornah et al., 2004; Penfield et al., 2004), respectively, were a kind gift from Professor Ian Graham (The University of York, UK). Seeds were sown on rockwool (Nitto Boseki), watered daily with 0.5 g L^−1^ Hyponex solution and grown under a 16/8 h light/dark cycle with white light from fluorescent lamps at approximately 50 µmol m^−2^ s^−1^ and 22°C. Sterilized seeds were sown on Suc-free Murashige and Skoog (MS) medium (Wako Pure Chemical) or MS medium with 2% (w/v) Suc where indicated, and solidified using 0.2% to 0.5% (w/v) gellan gum (Murashige and Skoog, 1962) to determine the effect of medium composition on phenotype. After sowing the seeds, the MS plates were stored at 4°C in the dark for 3 days. Next, the seedlings were grown in the light (as indicated above) for the indicated periods of time.

### Mutant Genotyping and Double-Mutant Generation

*fugu5-1* was used as a representative mutant allele of H^+^-PPase (Ferjani et al., 2011). dCAPS primers with one mismatch (fugu5-1-FW: 5′-CAGGCTGGTGTATCAGAGCAT-3′ and fugu5-1-RV: 5′-GACTCAACAGCCATGAGCTT-3′) were used for *fugu5-1* genotyping. PCR amplification followed by *Sph*I digestion was used to distinguish between the WT (134 bp + 18 bp fragments) and *fugu5-1* mutant (152 bp fragment). *icl-2*, *mls-2*, and *pck1-2* were genotyped as described previously (Eastmond et al., 2000; Cornah et al., 2004; Penfield et al., 2004). *fugu5-1*, *icl-2*, *mls-2*, and *pck1-2* plants were crossed with *an-1* mutant, which has been previously characterized (Kim et al., 2002; Minamisawa et al., 2011), to generate double mutants, and the genotypes of the F_2_ plants were checked using the above PCR-based markers.

### Morphological Observations and Cellular Phenotypic Analyses

Photographs of the gross plant phenotypes at 10 days after sowing (DAS) were obtained using a stereoscopic microscope (M165FC; Leica Microsystems) connected to a charge-coupled device (CCD) camera (DFC300FX; Leica Microsystems) and those at 21 DAS were obtained using a digital camera (D5000 Nikkor lens AF-S Micro Nikkor 60 mm; Nikon). Leaves were fixed in formalin/acetic acid/alcohol and cleared with chloral solution (200 g of chloral hydrate, 20 g of glycerol, and 50 mL of deionized water) to measure leaf area and cell number, as described previously (Tsuge et al., 1996). Whole leaves were observed using a stereoscopic microscope equipped with a CCD camera. Leaf palisade tissue cells were observed and photographed under a light microscope (DM-2500; Leica Microsystems) equipped with Nomarski differential interference contrast optics and a CCD camera. Cell size was determined as mean palisade cell area, determined from a paradermal view, as described previously (Ferjani et al., 2011).

### Observation and Quantitative Analysis of Epidermal Cells

For scanning electron microscopy (SEM), cotyledons were dissected from plants at the indicated growth stages. Samples were fixed overnight in formalin–acetic acid–alcohol (FAA; 4% formalin, 5% acetic acid, and 50% ethanol) at room temperature. The fixed specimens were dehydrated in an ethanol series [50%, 60%, 70%, 80%, 90%, 95%, 99.5%, and 100% (v/v); 60 min per step] and stored overnight in 100% (v/v) ethanol at room temperature. The ethanol was replaced with 3-methylbutyl acetate and the samples were dried in a critical-point dryer (JCPD-5; JEOL), sputter-coated with platinum–palladium using an anion sputter (JFC-1100; JEOL), and examined under an S-3400N SEM (Hitachi), as described previously (Maeda et al., 2014). SEM images of the adaxial side of cotyledons were used to quantify pavement cell complexity. The area and perimeter of individual pavement cells (25 cells from one cotyledon; six cotyledons total) were measured using ImageJ (version 1.63), and their complexity was quantified by calculating the undulation index (UI) (Thomas et al., 2003) using the following equation (Kürschner, 1997):

(1) $$UI=\frac{P}{2\pi \sqrt{A/\pi }}$$

where UI (dimensionless) is the undulation index, *P* (µm) is the cell perimeter, and *A* (µm^2^) is the cell area. Note that increased undulation index means increased pavement cell complexity, and *vice versa*.

### Histological Cross-Sections

For histological cross-sections, cotyledons were dissected (as indicated above), fixed overnight in FAA at room temperature, dehydrated in a graded series of ethanol [50%, 60%, 70%, 80%, 90%, and 95% (v/v); 30 min per step], and stored overnight in 99.5% (v/v) ethanol at room temperature. Next, fixed specimens were embedded in Technovit resin (Kulzer and Co.) according to the manufacturer's instructions and sectioned using a microtome (RM2125 RTS; Leica Microsystems). The sections were stained with toluidine blue and photographed under a microscope (BX-50; Olympus).

### Tubulin Polymerization Assay *in Vitro*

Tubulin polymerization was induced in a solution of 5 mg/mL porcine tubulin (T240, Cytoskeleton, Inc.), 80 mM piperazine-N,N′-bis(2-ethanesulfonic acid) (PIPES), 1 mM ethylene glycol-bis(β-aminoethyl ether)-N,N,N′,N′-tetraacetic acid, 2 mM MgCl_2_, 10% glycerol, 1 mM GTP, and 0, 1, 5, or 10 mM sodium pyrophosphate tetrabasic decahydrate (Sigma). The 20 µL reaction mixture was transferred into a 384 microplate (#242757, Thermo Scientific), and the absorption at 350 nm was measured every 30 s for 40 min at 37°C using a Multiskan FC plate reader (Thermo Scientific).

### Inhibitor Treatment

Seedlings were grown on Suc-free half-strength MS agar containing 2.3 g/L MS salt mix, 0.5 mg/L nicotinic acid, 0.1 mg/L thiamine HCl, 0.5 mg/L pyridoxine HCl, 0.1 g/L myo-Inositol, and 2 mg/L glycine (pH 5.8) at 22°C under constant light. Four-day-old seedlings were transferred into water containing 2 μM oryzalin (Wako) and incubated at 22°C in the dark for 2 h.

### Confocal Microscopy of Cortical MTs

The epidermis of the cotyledon was observed under an inverted fluorescence microscope (IX83-ZDC, Olympus) fitted with a confocal unit (CSU-W1, Yokogawa), an EM-CCD camera (iXon3-888, Andor), an UPLANSAPO 60× water-immersion objective (NA = 1.20, Olympus), and laser lines set at 488 and 561 nm. Images were acquired using MetaMorph software (Molecular Devices).

### Image Analysis for the Quantification of MT Dynamics

To quantify MT length and density, *z*-stack images at 2 μm intervals were acquired and analyzed using ImageJ software (https://imagej.nih.gov/ij/). The image stacks were projected to single images by maximum intensity projection. A 50 × 50-pixel region was collected from each cell and denoised using the Subtract background (5-pixel diameter) and Despeckle commands. Next, MT regions were extracted and converted to binary images using the Ridge detection plug-in (Steger, 1998). The total area and mean length of MTs were determined using the Analyze particle function.

To quantify the MT growth rate, time-lapse images at 5 s intervals were acquired and analyzed using ImageJ software. To extract the growing region of MTs, the three-frame shifted time-lapse images were subtracted from the original time-lapse images. The subtracted images were denoised by a Gaussian filter (σ = 1). The trajectory of MT growing regions was analyzed using the TrackMate plugin (Tinevez et al., 2016). The MT growing regions were detected using a LoG detector and filtered by a mean intensity filter. Tracking of MT growing regions was performed using Simple LAP tracker with the Track duration and Track displacement filters. Values of estimated blob diameter, linking max distance, gap-closing max distance, and gap-closing max frame gap were set at 10, 8, 8, 2, respectively.

### Mechanical Buckling Model

We constructed a two-dimensional model in which cell shapes were represented by particles of different sizes with interaction forces. **Figure S10A** shows a schematic representation of the initial geometry of the model, with a reduced number of particles for simplicity. Mesophyll cells are represented by green particles. Pavement and guard cells are shown as pairs of particles in blue and red, respectively, in the initial geometry. An oval covering a pair of particles was defined as the shape of the epidermal cell, as depicted in **Figure 7**.

It was assumed that each particle interacts with its adjacent neighbors. The interaction force was described as follows:

(2) $$\begin{array}{l} F_{i,j}^{int}=k^{int}f\left(\frac{d_{i,j}^{}}{d_{i,j}^{0}}\right)\frac{\left(x_{i}-x_{j}\right)}{\left|x_{i}-x_{j}\right|}\\ f\left(\xi \right)=\{\begin{array}{c} \xi^{\lambda }+c\;\xi^{-\lambda }\;-1-c\;\;\;\;\text{for}\;0<\xi \leq 1\\ \lambda \left(1-\text{c}\right)\left(\xi -1\right)\;\;\;\;\;\;\;\;\;\;\text{for}\;\xi >1\;\;\;\;\;\;\;\; \end{array}\;,\;\; \end{array}$$

where $F_{i,j}^{int}$ is the force working on the *i*-th particle caused by the interaction between the *i*-th and *j*-th particles. The parameter *k^int^* defines the strength of the particle interaction, and *c* and *λ* determine its dependence on the particle distance. The vector **x** *_i_* is a non-dimensional position vector of the center of the *i*-th particle. The distance between the *i*-th and *j*-th particles was calculated as *d_i,j_* = |**x** *_i_* − **x** *_j_*|, and its optimal value was defined as $d_{i,j}^{0}=r_{i}+r_{j}$, where *r_i_* is the radius of the *i*-th particle. The function $f\left(d_{i,j}^{}/d_{i,j}^{0}\right)$ takes positive and negative values when $d_{i,j}^{}/d_{i,j}^{0}<1$ and $d_{i,j}^{}/d_{i,j}^{0}>1$, which correspond to repulsive and attractive interactions, respectively (**Figure S10B**). The links of adjacent neighbors were determined from the initial geometry and were sustained throughout the calculation. Exceptionally, the guard cells were assumed not to bind to any mesophyll cells. It was also assumed that close approach of cells not defined as adjacent neighbors exerts a repulsive force as follows:

(3) $$\begin{array}{l} F_{i,j}^{rep}=k^{rep}g\left(\frac{d_{\text{i},\text{j}}^{}}{d_{i,j}^{0}}\right)\frac{\left(x_{i}-x_{j}\right)}{\left|x_{i}-x_{j}\right|}\\ g\left(\xi \right)=\{\begin{array}{c} \xi^{\lambda }+c\;\xi^{-\lambda }\;-1-c\;\;\;\;\text{for}\;0<\xi \leq 1\\ 0\;\;\;\;\;\;\;\;\;\;\;\;\;\;\;\;\;\;\;\;\;\;\;\;\;\;\;\;\;\;\;\;\;\;\;\;\;\;\text{for}\;\xi >1.\;\;\;\;\;\;\;\; \end{array}\;\;\;\;\;\; \end{array}$$

The radii of mesophyll-representing particles were assumed to have a continuous uniform distribution on the interval (0.7, 1.3). Cellular growth was described by increasing the radius of particles by 28% in 40,000 time-steps. The radii of guard cell-representing particles were set at *r_i_*= 0.2 and maintained constant.

The pavement cell was represented by two particles of initial radius *r_i_*= 0.3. Growth of the pavement cells was described as extension of these cells at constant height (**Figure S10D**). For that purpose, a new particle of radius *r_i_*= 0.03 was inserted in the middle of the cell (between two particles) at time *t* = 1. The radii of new particles were gradually increased to 0.3 in 40,000 time-steps.

The epidermal layer, which is described as the sequence of particles representing pavement and guard cells, was assumed to have bending rigidity to express the smooth surface of the leaf (**Figure S10C**). The force caused by the bending rigidity was introduced as follows:

(4) $$F_{i}^{bend}=k^{bend}\left(\frac{\theta_{i}-\theta_{i-1}}{d_{i,i-1}}n_{i-1,i}+\frac{\theta_{i}-\theta_{i+1}}{d_{i,i+1}}n_{i,i+1}\right),$$

where *θ*_*i*_ is the angle between the vectors **x** *_i_*-**x** *_i_*_-1_ and **x** *_i_*_+1_ **-x** *_i_*, *k^bend^* defines the strength, and **n** *_i_*_-1,_ *_i_* is the unit vector normal to the vector **x** *_i_*-**x** *_i_*_-1_ (π/2-rotation).

Taken together, the sum of the force contributing to movement of the *i*-th particle was assigned as follows:

(5) $$F_{i}=\sum_{j}F_{i,j}^{int}+\sum_{j}F_{i,j}^{rep}+\;F_{i}^{bend}.$$

The motion of the particle was described as follows:

(6) $$x_{i}^{t+1}=x_{i}^{t}+\gamma^{-1}F_{i}\Delta t+z,$$

where *γ* ^−1^ ***F***_*i*_ corresponds to the velocity of the *i*-th particle, *γ* corresponds to the friction coefficient, and *t* represents the time step. The random vector **z** was introduced to prevent accidental generation of a gap between particles in the numerical simulations.

The positions of the particles at both ends of the epidermal layer were independent from Eq. (5). The tissue width was controlled by moving the particles outward (WT) or by fixing them at their initial positions (*an-1 fugu5-1* mutant). For the WT, the tissue width was increased by 28% in 40,000 time-steps.

The development of particle behaviors was calculated using C++. In this study, *c* = 1.63, λ = 2, *k^rep^* = 30, *k^bend^* = 25, and *γ* = 20. Interactions among mesophyll particles were dependent on *k^int^* = 30, that between mesophyll and pavement particles on *k^int^* = 20, and among epidermal particles on *k^int^* = 70.

### Statistical Analysis

To quantify pavement cell phenotype, data from at least five cotyledons (>115 pavement cells) were used to calculate the undulation index, and the values were subjected to statistical analyses using tow-tailed Student's *t* test with Bonferroni correction, or ANOVA with Tukey's honestly significant difference (HSD) test, KaleidaGraph ver. 4.1.4. To quantify pavement cell neck width and lobe length, 825 measurements from five cotyledons were averaged and the values were subjected to statistical analyses using Student's *t* test. To quantify MTs length and density, data from 120 pavement cells were averaged and the values were subjected to statistical analyses using ANOVA with Scheffe test. Finally, to quantify the growth rate of cortical MTs, data from 30 cells (80 < MTs/cell) were averaged and subjected to statistical analyses using ANOVA with Scheffe test.

## Results

### Excess PPi Reduced Pavement Cell Complexity

**Figure 1:**
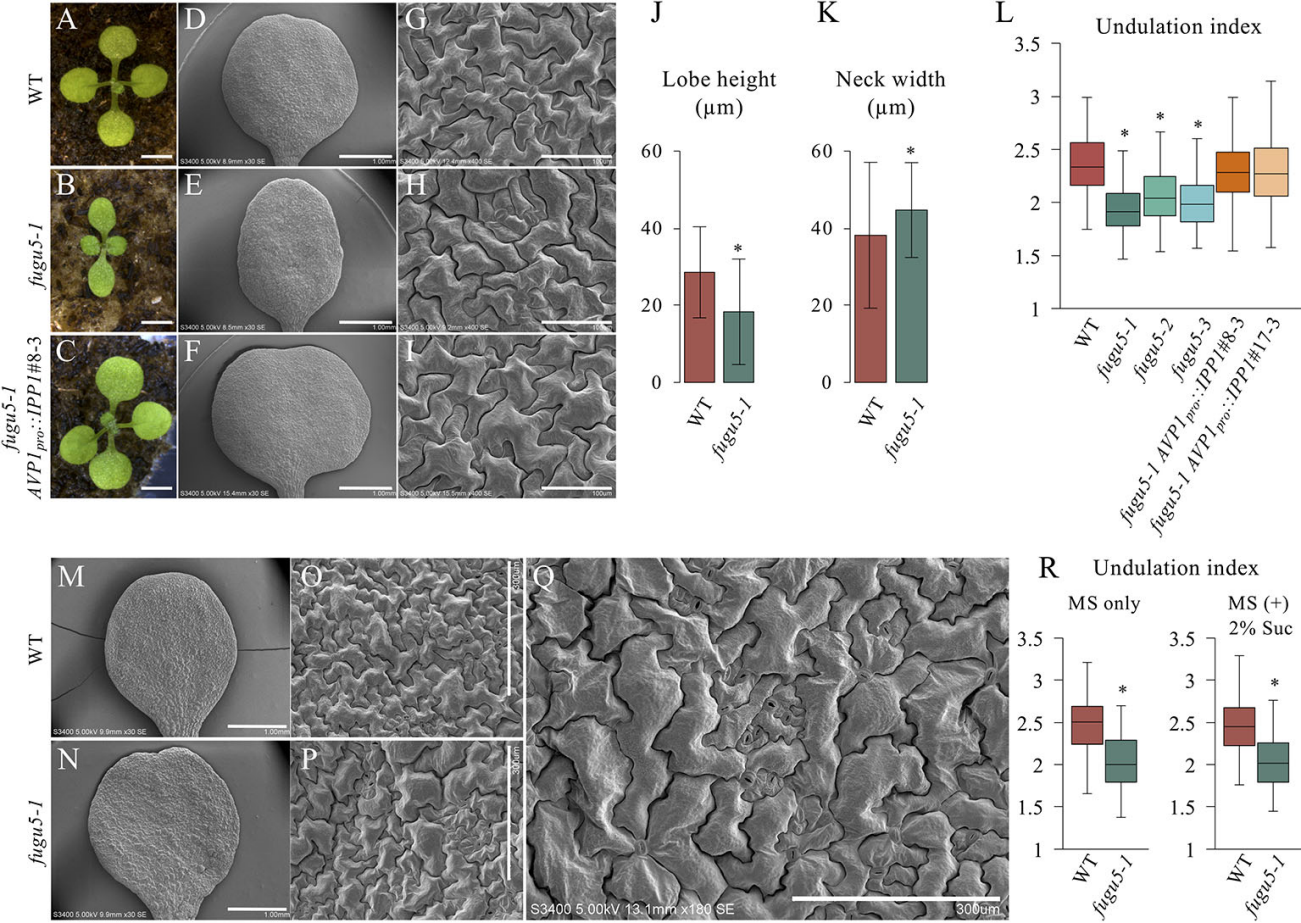
Analysis of adaxial pavement cell phenotype. **(A**–**C)** Photographs of seedlings of the indicated genotypes at 14 DAS. Scale bar, 2 mm. **(D**–**F)** SEM images of the adaxial side of cotyledons at 25 DAS. Scale bar, 1 mm. **(G**–**I)** Magnified SEM images of pavement cells on the adaxial side of cotyledons. Scale bar, 100 µm. **(J)** Average neck width and lobe length **(K)** of pavement cells (*n* = 825 measurements of five cotyledons). Data are means ± standard deviation (SD) at *P <* 6.6 × 10^−57^ (lobe), *P <* 1.0 × 10^−16^ (neck) between the WT and *fugu5-1* (two-tailed Student's *t* test). **(L)** Undulation index (*n* = 150 pavement cells, taken from six cotyledons). Data are means ± SD. Asterisk represents a significant difference at *P* < 1.6 × 10^−16^ compared to the WT (two-tailed Student's *t* test, Bonferroni correction). **(M**, **N)** SEM images of WT and *fugu5-1* cotyledons, respectively, grown for 20 DAS on MS plates containing 2% sucrose. Scale bar, 1 mm. **(O**, **P)** SEM images of adaxial side pavement cells of the WT and *fugu5-1* mutant, respectively. Scale bar, 300 µm. **(Q)** Magnified SEM image of *fugu5-1* adaxial pavement cells. Scale bar, 300 µm. **(R)** Undulation index (*n* = 125 pavement cells from at least five cotyledons). Data are means ± SD. Asterisk represents a significant difference between the two genotypes at *P <* 1.0 × 10^−20^ (two-tailed Student's *t* test). DAS, days after sowing. Suc, sucrose.

*fugu5* mutants show a strong phenotype in their cotyledons, and so were used as model organs. The adaxial side of mature cotyledons was investigated by SEM, which revealed that pavement cells are less complex in the *fugu5* mutant compared to the WT (**Figures 1A–I**). Quantification of lobe height and neck width revealed that *fugu5-1* has shallower lobes and wider necks than the WT (**Figures 1J, K**). Consistently, the UI in all three *fugu5* alleles was inferior to that of the WT (**Figure 1L**). Importantly, all *fugu5* phenotypes recovered in the *fugu5-1 AVP1_pro_::IPP1* lines, in which PPi was specifically removed from the *fugu5* background (**Figures 1A–I, L**).

We reported that there are fewer and larger palisade tissue cells in *fugu5*, the so-called compensation phenotype (Ferjani et al., 2007; Ferjani et al., 2008; Ferjani et al., 2011). Excess PPi inhibits gluconeogenesis during seedling establishment, and compensation is complemented by hydrolyzing PPi or exogenous Suc supply (Ferjani et al., 2011; Ferjani et al., 2012). Unexpectedly, Suc supply did not restore the shape of pavement cells in *fugu5* (**Figures 1M–R**). Together, these results suggest that excess PPi exerts different effects on the palisade and epidermis.

### Excess PPi Altered Pavement Cell Size and Distribution

**Figure 2:**
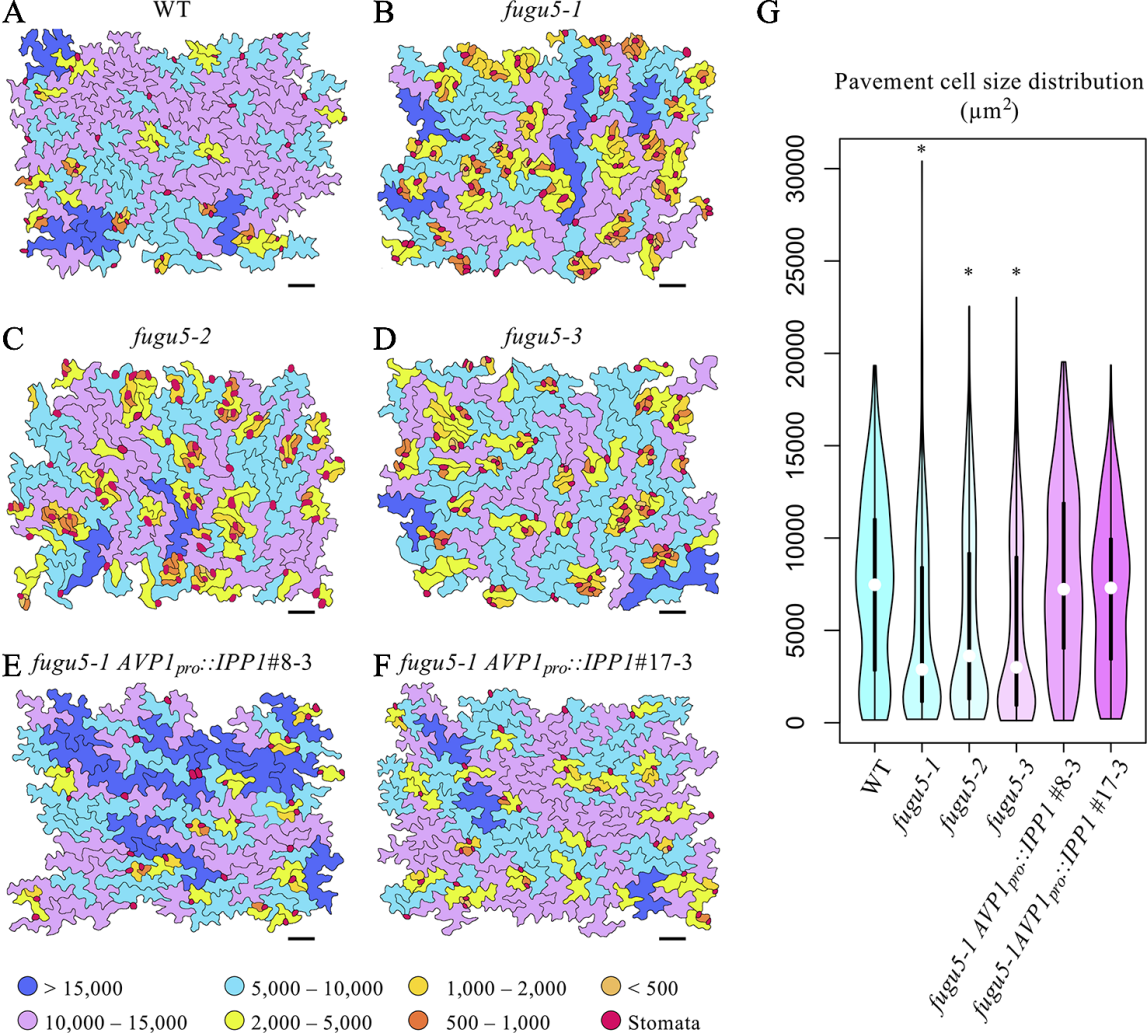
Distribution of pavement cell size. **(A**–**F)** SEM images of adaxial side pavement cells of the cotyledons of WT, *fugu5-1*, *fugu5-2*, *fugu5-3*, *fugu5-1 AVP1_pro_::IPP1*#8-3, and *fugu5-1 AVP1_pro_::IPP1*#17-3, respectively. Scale bar, 100 µm. Cells were traced and color-coded based on their size (µm^2^, see color scale chart). Note that the *fugu5* mutants have a disturbed stomatal one-cell-spacing rule. **(G)** Violin plot showing pavement cell size distribution (*n* > 200 pavement cells from three cotyledons). Data are means ± SD. Asterisk represents a significant difference compared to the WT at *P* < 0.0001 (ANOVA with Tukey HSD test, KaleidaGraph ver. 4.1.4).

To gain insight into the effect of excess PPi on pavement cells, SEM images of the adaxial side of the cotyledon were traced and color-coded based on their size (**Figure 2**). WT pavement cells were not only more complex but also significantly larger than those of the *fugu5* mutants (**Figures 2A–D, G**). In addition, small pavement cells in *fugu5* mutants tended to form small clusters with stomata on the adaxial side of cotyledons (compare **Figure 2A** to **Figures 2B–D**). Consistently, stomatal distribution was aberrant in all *fugu5* mutant alleles, in violation of the one-cell-spacing rule (**Figures 2B–D**; see also Asaoka et al., 2019). Pavement cell size and complexity and the stomatal distribution recovered when PPi was specifically removed from the *fugu5-1 AVP1_pro_::IPP1*#8-3 and *fugu5-1 AVP1_pro_::IPP1*#17-3 lines (**Figures 2E–G**).

### Cotyledon Development and Pavement Cell Phenotype Are Synergistically Enhanced in *an-1 fugu5-1*

**Figure 3:**
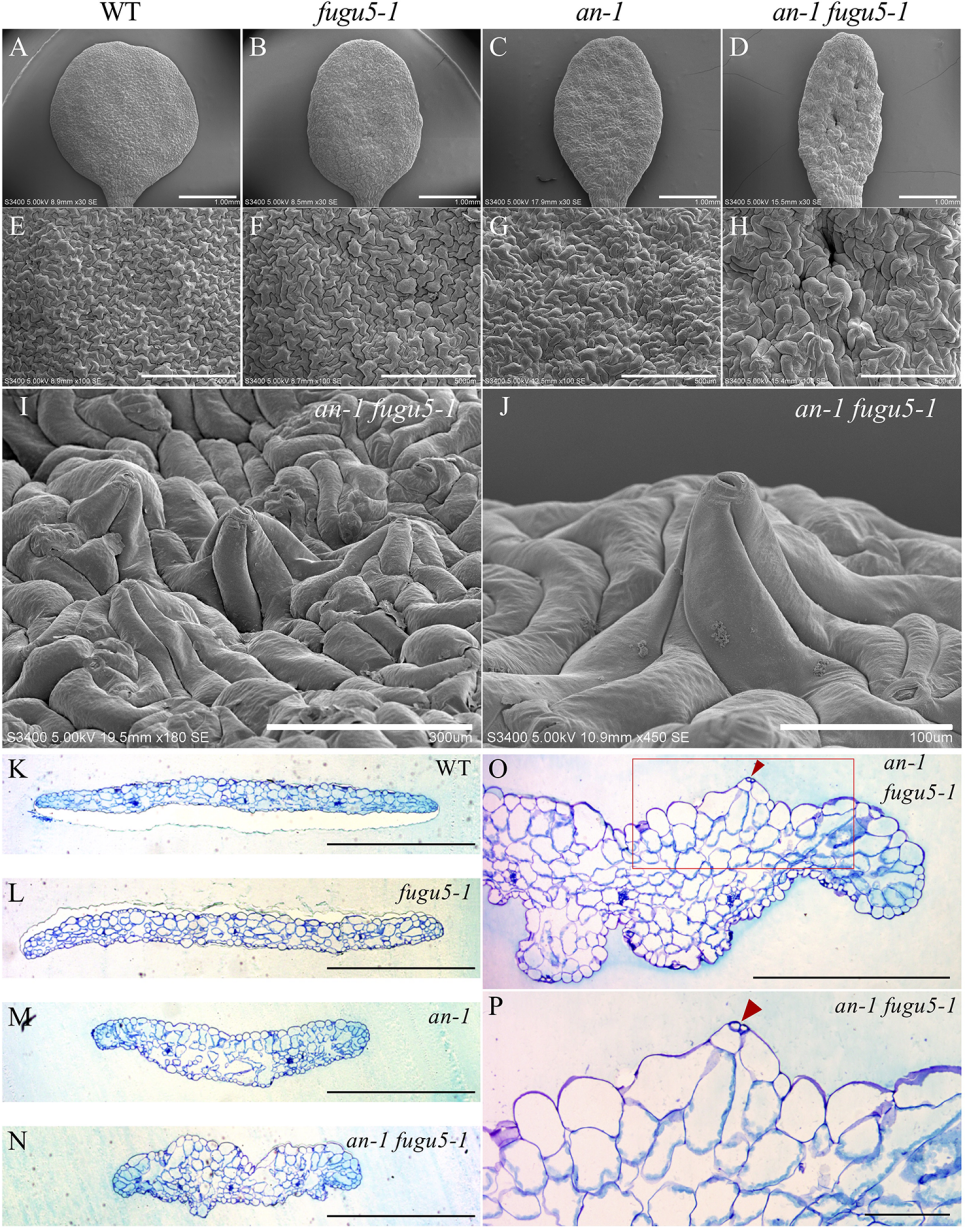
Observation of adaxial epidermis developmental defects in *an-1 fugu5-1*. **(A**–**D)** SEM images of the adaxial side of the cotyledon of the WT, *fugu5-1*, *an-1*, and *an-1 fugu5-1*, respectively, at 25 DAS. Scale bar, 1 mm. **(E**–**H)** SEM images of the adaxial side pavement cells of the WT, *fugu5-1*, *an-1*, and *an-1 fugu5-1*, respectively. Scale bar, 500 µm. **(I**, **J)** SEM images of the adaxial side of the cotyledon of *an-1 fugu5-1* showing developmental defects of pavement cells. Scale bar, 300 µm **(I)**, 100 µm **(J)**. **(K**–**N)** Histological cross sections of the WT and *fugu5-1*, *an-1*, and *an-1 fugu5-1* mutant cotyledons at 25 DAS. Samples were stained with toluidine blue. Scale bar, 1 mm. **(O**, **P)** Magnified cross-sectional images of *an-1 fugu5-1.* Scale bar, 500 µm. **(O)** Aberrant pavement cells in *an-1 fugu5-1* (red box in (O)), are magnified in **(P)**. Scale bar, 100 µm. Red arrowheads indicate stomata.

ANGUSTIFOLIA (AN) is the sole homolog of CtBP/BARS in Arabidopsis. The CtBP/BARS protein family has diverse functions, intercellular localization, and developmental role in a wide range of land plants (Kim et al., 2002; Minamisawa et al., 2011; Furuya et al., 2018; Hashida et al., 2019). AN regulates the width of Arabidopsis leaves by controlling the polar elongation of leaf cells. In fact, the *an-1* mutant displayed an abnormal arrangement of cortical MTs in leaf cells, which accounted for its abnormal cell shape (Kim et al., 2002). Therefore, AN might regulate the polarity of cell growth by controlling the arrangement of cortical MTs (Kim et al., 2002). The AN-DYRKP complex reportedly regulates the alignment of actin filaments during centripetal nuclear positioning in leaf cells (Iwabuchi et al., 2019). Interestingly, the typical oblong cotyledons of *fugu5-1* mutants are reminiscent of the *an-1* mutant (Kim et al., 2002; Minamisawa et al., 2011). Thus, to gain insight into the genetic interaction between AN and H^+^-PPase, we constructed *an-1 fugu5-1* double mutants and analyzed their pavement cell phenotype (**Figure 3**). Surprisingly, the *an-1 fugu5-1* double mutants displayed a drastic phenotype, consisting of extremely narrow cotyledons and a wavy epidermis (**Figures 3A–D**). SEM of the adaxial side of *an-1 fugu5-1* cotyledons revealed 3D protrusions whereby twisted pavement cells lifted a stoma (**Figures 3E–J**). In addition, histological cross sections confirmed the above SEM observations and further showed that the wavy-epidermis phenotype occurred on both sides of cotyledons (**Figures 3K–N**; **Figure S1**). Magnified images of histological cross sections confirmed that a pair of guard cells was indeed lifted atop the protruding pavement cells (**Figures 3O, P**).

### Cotyledon Expansion Is Severely Restricted Along the Mediolateral Axis in *an-1 fugu5-1*

**Figure 4:**
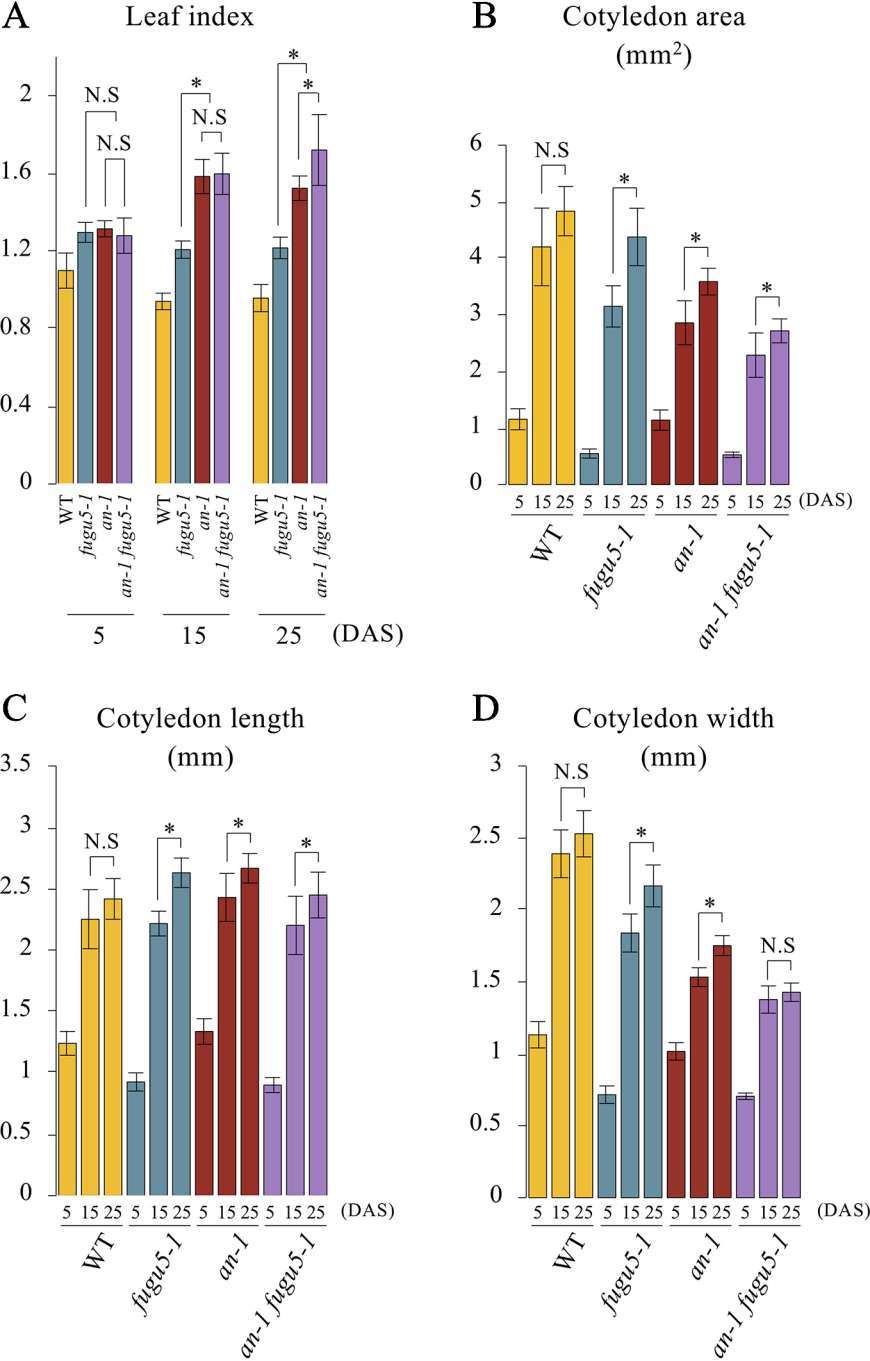
Quantitative time-course analysis of cotyledon morphological phenotypes. **(A)** Leaf index was determined for the WT, *fugu5-1*, *an-1*, and *an-1 fugu5-1* at the indicated growth stages. **(B)** The cotyledon area was determined for each genotype at the indicated growth stages. **(C**, **D)** The cotyledon length and width were determined for each genotype at the indicated growth stages. Values were calculated from SEM images of a time-course analysis of each genotype (**Figure S4**). Data are means ± SD (WT, *fugu5-1*, *an-1*: *n* = 6; *an-1 fugu5-1*: *n* = 5). Asterisk represents a significant difference between genotypes at *P <* 0.05 (two-tailed Student's *t* test). DAS, days after sowing. N.S, not significant.

Based on the above results, we examined the gross morphological phenotype of *an-1 fugu5-1* to evaluate the wavy-epidermis phenotype. We performed a quantitative time-course analysis of various morphological features of cotyledons at three distinct developmental stages. The leaf index (LI = the ratio of leaf blade length to leaf blade width) values indicated that while WT cotyledons were nearly spherical (LI ≒ 0.9–1), those of *fugu5-1* were slightly longer than wide (LI ≒ 1.2), *an-1* were more elongated than *fugu5-1* (LI = 1.3–1.5), and *an-1 fugu5-1* were the most elongated (LI = 1.3–1.7) (**Figure 4A**). The differences among the above genotypes were small at early stages of cotyledon development (5 DAS), but greater at later stages (15 and 25 DAS). Quantification of cotyledon area, length, and width confirmed that *an-1 fugu5-1* cotyledon expansion along the medio-lateral axis was arrested at 15 DAS or earlier (**Figures 4B–D**).

To determine the timing of emergence of the 3D-growth phenotype, whole *an-1 fugu5-1* seedlings were collected at 5 and 10 DAS, fixed, and prepared for SEM. The 3D-growth phenotype was not visible at 5 DAS on the adaxial side of cotyledons (**Figure S2A**). However, at 10 DAS, the adaxial side of *an-1 fugu5-1* cotyledons displayed a wavy texture, typical 3D growth not evident in the parental lines (**Figures S2B, S3**, and **S4**). Together, the above results indicated that the drastic developmental defects in *an-1 fugu5-1* occur at an early post-germinative developmental stage, and that they are enhanced as organ maturation progresses.

### Defects in Pavement Cell Differentiation Are Specifically Triggered by Excess PPi

As mentioned above, while exogenous supply of Suc rescued the *fugu5* palisade tissue phenotype, epidermis developmental defects did not recover unless PPi was specifically removed, such as in the *fugu5-1 AVP1_pro_::IPP1*#8-3 and *fugu5-1 AVP1_pro_::IPP1*#17-3 lines (**Figure 1**). Consistently, Suc availability did not affect the *an-1 fugu5-1* phenotypes, as indicated by the formation of 3D protrusions on the adaxial side of *an-1 fugu5-1* grown for 20 days on MS medium containing 2% Suc (**Figures S5, S6**). We previously reported that mutants with defects in gluconeogenesis (*pck1-2*) or the glyoxylate cycle (*icl-2*; *mls-2*), produced less Suc from TAG and exhibited compensation in their cotyledons that recovered upon Suc supply (Takahashi et al., 2017). This led to the conclusion that lowered Suc production, but not excess PPi, is necessary to trigger compensation in palisade tissue (Takahashi et al., 2017). However, the epidermal cells of the above mutant lines were not examined.

**Figure 5:**
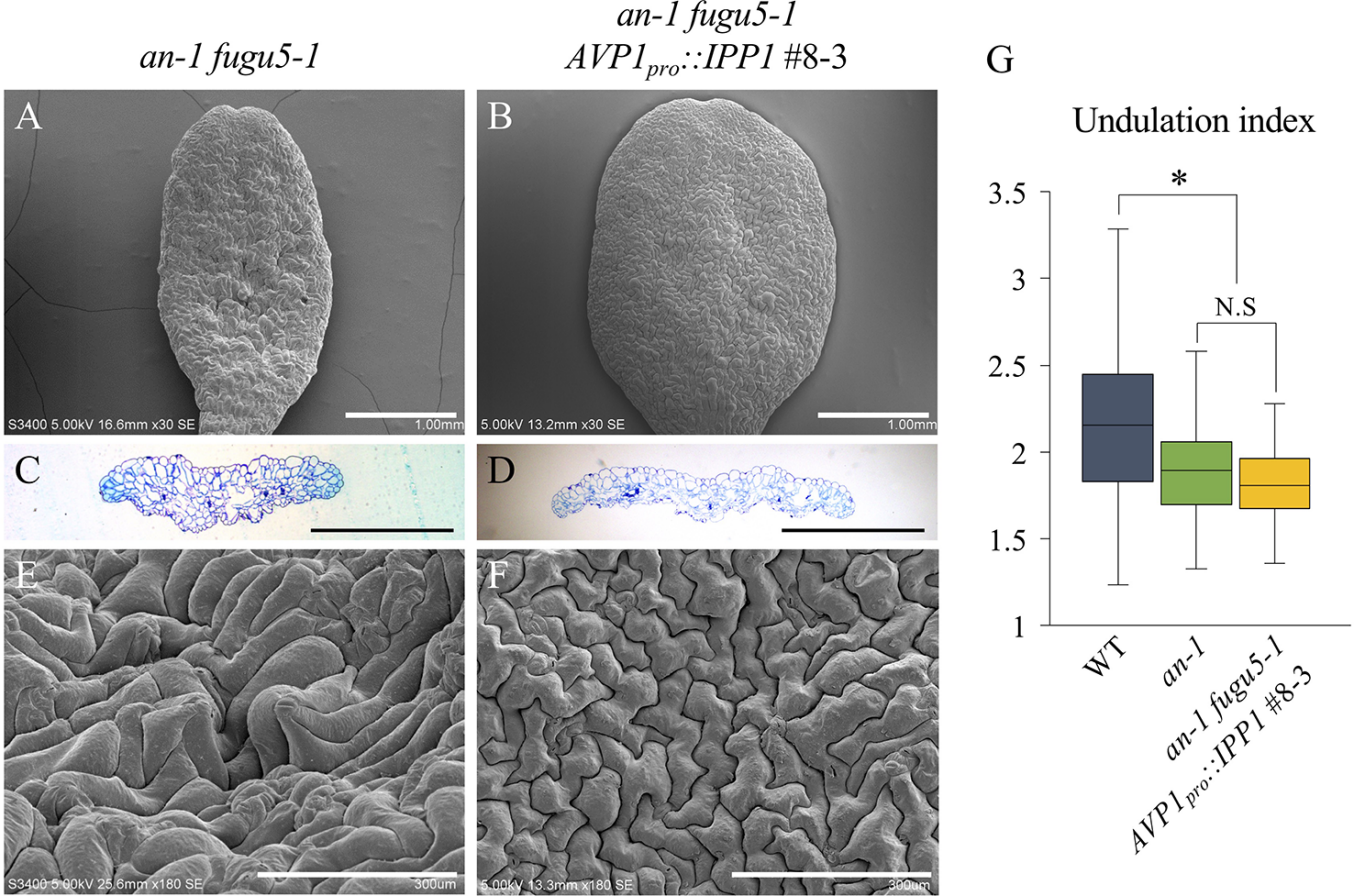
Expression of *IPP1* rescued *an-1 fugu5-1* developmental defects. **(A**, **B)** SEM images of the adaxial side of cotyledons of *an-1 fugu5-1* and *an-1 fugu5-1 AVP1_pro_::IPP1* #8-3 at 25 DAS. Scale bar, 1 mm. **(C**, **D)** Cotyledon histological cross sections of *an-1 fugu5-1* and *an-1 fugu5-1 AVP1_pro_::IPP1* #8-3 at 25 DAS. Scale bar, 1 mm. **(E**, **F)** Magnified SEM images of the adaxial side of cotyledons of *an-1 fugu5-1* and *an-1 fugu5-1 AVP1_pro_::IPP1* #8-3 at 25 DAS. Note that *an-1 fugu5-1* developmental defects of the epidermis *were* recovered by expression of *IPP1*. Scale bar, 300 µm. **(G)** Undulation index of pavement cells (*n* = 115 pavement cells from at least four cotyledons). Data are means ± SD. Asterisk represents a significant difference compared to the WT at *P <* 0.0001 (ANOVA with Tukey HSD test, KaleidaGraph ver. 4.1.4). DAS, days after sowing. N.S, not significant.

The pavement cells of *icl-2*, *mls-2*, and *pck1-2* were indistinguishable from the WT in terms of their UI values (**Figures S7A–G**). Next, we constructed *an-1 icl-2*, *an-1 mls-2*, and *an-1 pck1-2* mutants and evaluated their pavement cell phenotypes. Importantly, while these double mutants displayed a severely reduced cotyledon width reminiscent of *an-1 fugu5-1*, they did not exhibit 3D protrusions (**Figures S7H–M**). In addition, the UI values of the above double mutants were comparable to that of *an-1* (**Figure S7N**). Together, these results confirmed that compensation in palisade tissue is due to Suc deficit, while the pavement cell patterning defects, including 3D protrusions, are PPi-dependent. If so, removal of PPi from the *an-1 fugu5-1* double mutant background would be sufficient to suppress the 3D protrusions. As expected, phenotypic analysis of *an-1 fugu5-1 AVP1_pro_::IPP1*#8-3 confirmed that PPi removal was indeed necessary and sufficient for restoring pavement cell patterning, which mimicked that of the *an-1* single mutant (**Figures 5A–F**). Quantification of the UI further supported these findings (**Figure 5G**).

### The H^+^-PPase Promotes MT Growth and Genetically Interacts With *AN* in MT Dynamics

**Figure 6:**
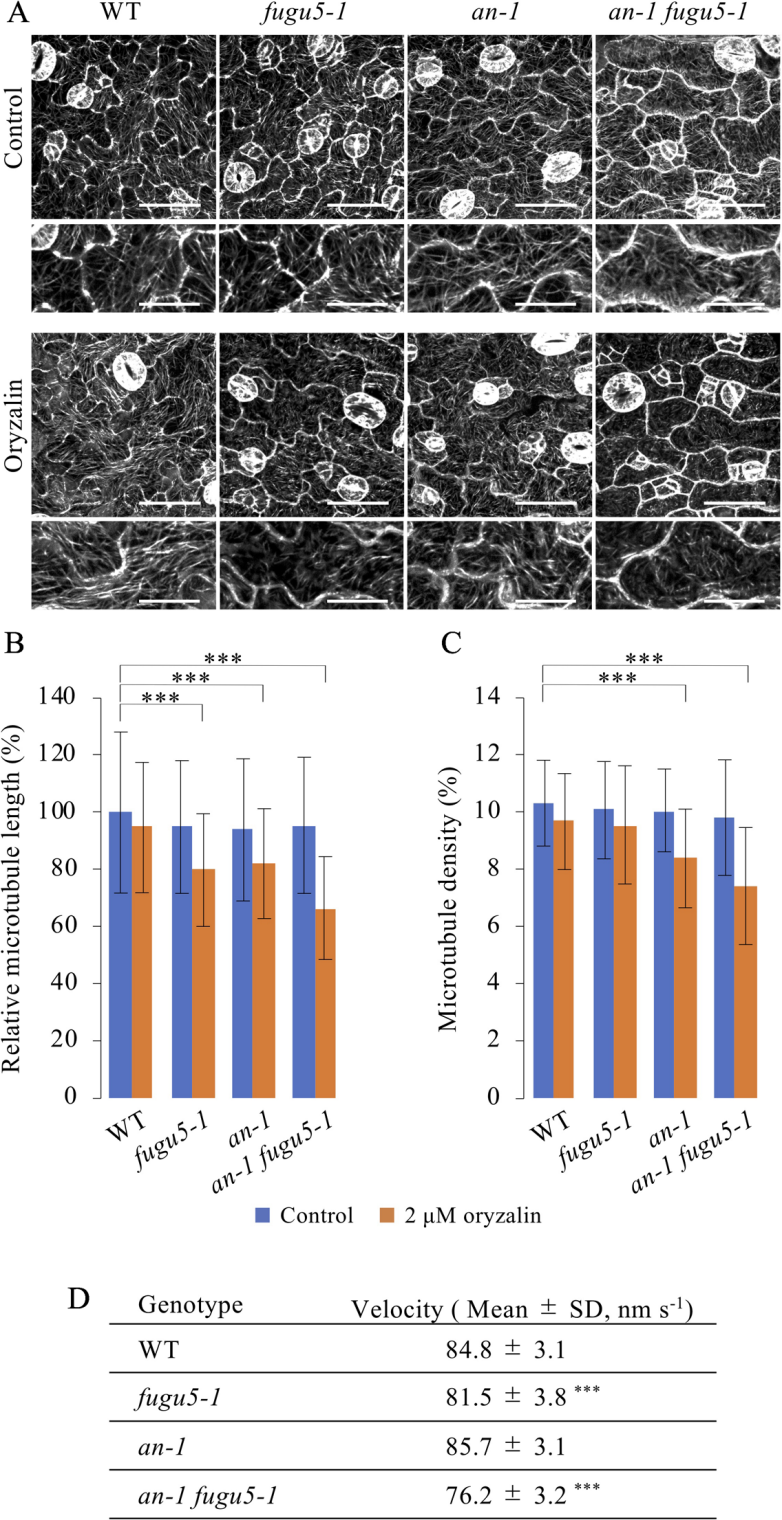
Effect of oryzalin on cortical microtubule dynamics in pavement cells. **(A)** Cortical microtubules in the cotyledon epidermis of WT, *fugu5-1*, *an-1*, and *an-1 fugu5-1* plants expressing *UBQ10::EYFP-TUB6*. Four-day-old seedlings were observed without (control) or after 2 h of treatment with 2 μM oryzalin (oryzalin). Bars, 50 μm. 200%-magnified close-ups are shown below each image. Bars, 25 μm. **(B**, **C)** Microtubule length **(B)** and density **(C)** of the cotyledon epidermis. Data are means ± SD (***P < 0.001, ANOVA with Scheffe test, *n* = 120 cells). **(D)** Growth rate of cortical microtubules in the cotyledon epidermis of WT, *fugu5-1*, *an-1*, and *an-1 fugu5-1* plants expressing *UBQ10::EYFP-TUB6*. Measurements were performed on 5-day-old seedlings. Data are means ± SD [***P < 0.01 to WT, ANOVA with Scheffe test, *n* = 30 cells (80 < MTs/cell)].

Given the axial role of MTs in the control of pavement cell morphogenesis (Fu et al., 2005), the above observations suggested that PPi affects MT dynamics. Thus, we investigated the role of MTs in the regulation of cell morphology in our system. First, we investigated MTs in the epidermis of cotyledons of the *fugu5-1*, *an-1*, and *an-1 fugu5-1* mutants. To observe MTs in living cotyledon epidermis, we crossed *UBQ10::EYFP-TUB6* plants (Sugiyama et al., 2017) with *fugu5-1*, *an-1*, and *an-1 fugu5-1* mutant plants and obtained homozygotes harboring *UBQ10::EYFP-TUB6.* In the pavement cells of young cotyledons from the *fugu5-1*, *an-1*, and *an-1 fugu5-1* mutants, cortical MTs were normally organized and indistinguishable from those in WT cells (**Figure 6A**). To examine the properties of MTs in these mutants, we treated the seedlings with 2 µM oryzalin, which is not sufficient to disrupt cortical MTs in WT plants. As expected, cortical MTs were intact in WT plants even after oryzalin treatment (**Figure 6A**). By contrast, in *fugu5-1*, *an-1*, and *an-1 fugu5-1* pavement cells, cortical MTs were slightly fragmented and sparse (**Figure 6A**). Indeed, the mean length of cortical MTs in *fugu5-1*, *an-1*, and *an-1 fugu5-1* cells was shorter than in WT cells (**Figure 6B**). The mean MT length was shortest in *an-1 fugu5-1* cells (**Figure 6B**). The density of cortical MTs, calculated as the area of EYFP-TUB6-positive pixels, was also lower in *an-1*, and markedly lower in *an-1 fugu5-1* cells, than in WT cells (**Figure 6C**). These results suggested that cortical MTs are more unstable in *fugu5-1* and in *an-1* cells than in WT cells and that genetic interactions of *FUGU5* and *AN* influence MT stability.

We next investigated the dynamics of cortical MTs in young pavement cells. Cortical MTs exhibit growth, shrink, rescue, catastrophe, and stop (Shaw et al., 2003). However, due to the high density of cortical MTs in young cotyledons, we could not analyze the dynamics of MTs. Instead, we examined the net growth rate of cortical MTs by subtracting the time-shifted images from the time-lapse images and capturing the track of their growth (**Movie S1**). Next, we quantified the net growth rate (velocity) of cortical MTs (**Figure 6D**). MT growth rate was slightly lower in *fugu5-1* cells and markedly lower in *an-1 fugu5-1* cells than in WT, again suggesting that *FUGU5* promotes MT growth and genetically interacts with *AN* to influence MT dynamics.

Because *fugu5-1* cells accumulate a high level of PPi in the cytosol (Ferjani et al., 2011), we reasoned that MT dynamics might be directly affected by excess PPi ions in *fugu5-1* mutants. To assess this possibility, we examined the tubulin polymerization rate *in vitro* in the presence of 1, 5, and 10 mM PPi (**Figure S8**). Consistently, addition of PPi led to a dose-dependent delay of tubulin polymerization (**Figure S8**). Thus, PPi at higher than physiological concentrations may directly interfere with MT dynamics by inhibiting their polymerization (Weiner et al., 1987; Heinonen, 2001). We also noticed that reduced pavement cell complexity and stomatal patterning defects were evident at 4 DAS (**Figure S9**).

### Three-Dimensional Protrusions in *an-1 fugu5-1* Are Caused by Mechanical Buckling Between Adjacent Pavement Cells

To examine the possibility that mechanical buckling of the epidermis generates the undulation seen in *an-1 fugu5-1*, a computational model of the mechanical interaction between cells was developed (**Figure S10**). We applied a model framework in which cells were represented by particles that grow and crowd each other, followed by a change in tissue shape (Takigawa-Imamura et al., 2015; Higaki et al., 2017). In this study, the cross-sectional structure of a part of the upper surface of a cotyledon was considered in a 2D space. The model comprised pavement cells, guard cells, and mesophyll cells, which were assigned abstract round shapes for simplicity (**Figure S10**). Repulsive interaction was assumed between cells to prevent overlaps, and an attractive interaction was assumed so that cell-cell connections were sustained (**Figure S10B**). The epidermal layer was assumed to have bending stiffness. To describe the smooth surface of the cotyledon, the restoring force due to bending stiffness was introduced along the sequence of pavement and guard cells (**Figure S10C**). The growth of mesophyll cells was described by increasing their diameter. The growth of pavement cells was described as horizontal extension at constant height (**Figure S10D**). Guard cell growth was omitted because the change in guard cell width is considered to be limited. In the model guard cells were distinguished from pavement cells in that they were not attached to the mesophyll and are less tall.

**Figure 7:**
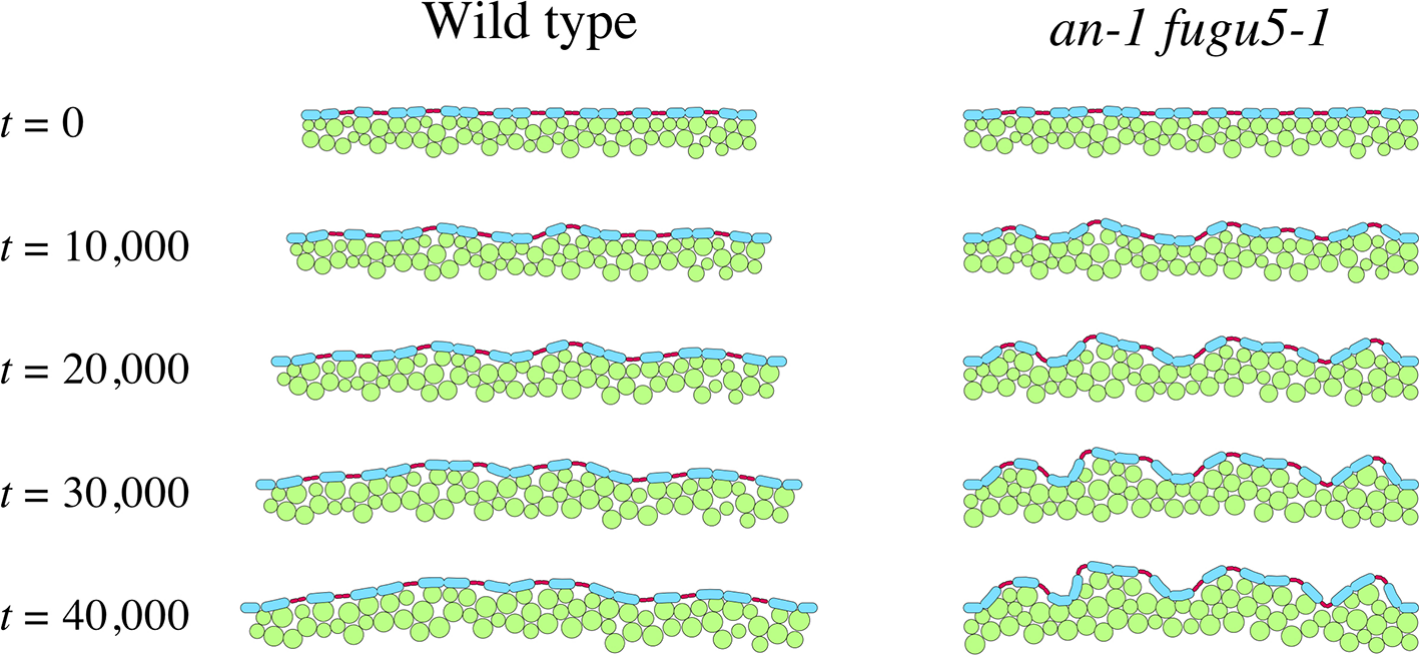
Effect of medio-lateral growth limitation on the epidermis. Computational model describing tissue growth of the WT (left panel) and the *an-1 fugu5-1* double mutant (right panel). Green circles denote mesophyll cells, and blue and red ovals represent pavement and guard cells, respectively. Mesophyll and pavement cells increased in size over time. The tissue width increased over time in the WT but remained constant in the *an-1 fugu5-1* double mutant.

**Figure 7** shows tissue growth described using the model (**Movie S2** and **S3**). We assumed that tissue width increases gradually in the WT, whereas it is constant in *an-1 fugu5-1* based on our observations (**Figure 4D**). The computational simulation showed that undulations of the epidermis emerged in *an-1 fugu5-1* but not in the WT as the pavement cells expanded (**Figure 7**), demonstrating that coordination between the growth orientation of the mesophyll and the expansion of the epidermis significantly affects tissue shape. Notably, peaks of undulating epidermis were mostly at the sites of guard cells, suggesting that attachment to the mesophyll suppresses mechanical buckling at the sites of pavement cells.

## Discussion

Cell differentiation is a focus of investigation, given the fundamental role of specialized cell types in the execution of vital functions at the cell, tissue, organ, and organismal levels. Although key transcription factors orchestrate cell fate at the transcriptional level, metabolism has also been proposed to be vital in cell differentiation. We evaluated the link between the role of H^+^-PPase in PPi homeostasis and its impact on plant development. Here, we present several lines of evidence on the role of metabolism in this process, with emphasis on PPi homeostasis and its multifaceted impact on developmental regulation of plant leaves, tissues, and cells.

### The Pleiotropic, Yet Specific, Targets of PPi in Arabidopsis

PPi is a metabolic byproduct (Heinonen, 2001), whose accumulation at high levels in cells of all kingdoms has been reported not only to inhibit anabolic reactions but also to cause lethality in extreme cases (Ferjani et al., 2011; Ferjani et al., 2014; Segami et al., 2018). Indeed, the H^+^-PPase loss-of-function *fugu5* mutants of Arabidopsis exhibit a pleiotropic phenotype, as they fail to reactivate cell cycling in cotyledonary palisade tissue following seed imbibition due to inhibition of gluconeogenesis from seed-stored TAG (Ferjani et al., 2011). The lack of Suc, the sole source of energy in the form of NTPs at a critical stage of the plant life cycle, triggered cellular phenotypes (compensation) and reduced hypocotyl elongation, both of which can be interpreted as metabolism-related developmental defects (Ferjani et al., 2011; Katano et al., 2016; Takahashi et al., 2017). Indeed, the addition of Suc exogenously, or the removal of PPi, restored the above phenotypes.

Moreover, excess PPi in guard cells inhibited stomatal closure, likely by perturbing its metabolism and/or omitting its responsiveness to the phytohormone abscisic acid (Asaoka et al., 2019). Therefore, PPi exerts an inhibitory effect on specific targets within different cell types. In other words, the function of H^+^-PPase, the major PPi-hydrolyzing enzyme in plant cells (Ferjani et al., 2014; Segami et al., 2018), goes beyond housekeeping, and the breadth and specificity of its contribution warrants investigation.

### Excess PPi Within Pavement Cells Reduces Their Size and Complexity

Given that PPi is produced in all plant cell types, particularly those with an active metabolism, we extended our investigation to the epidermis, a major tissue that wraps the whole plant body. Pavement cells in *fugu5* were less complex in shape, which was not recovered by exogenous Suc supply. This finding suggested that the reduced pavement cell complexity in *fugu5* is not a result of reduced Suc production (due to inhibited gluconeogenesis) but is triggered by excess PPi. In support of this interpretation, pavement cell complexity was restored when PPi was specifically hydrolyzed by the soluble PPase IPP1 in the *fugu5* background (**Figure 1L**). Moreover, pavement cells were as complex as the WT even in mutants with defects in gluconeogenesis (*pck1-2*) or the glyoxylate cycle (*icl-2* and *mls-2*), all of which exhibited severely reduced TAG-Suc conversion during seedling establishment (**Figure S7G**; Takahashi et al., 2017). Taken together, the above results indicated that excess PPi targets likely differ even among adjacent leaf tissues, namely the epidermis and the palisade tissues. We recently reported that UGPase is the major target of the inhibitory effect of excess PPi *in vivo* (Ferjani et al., 2018). At this stage, while the partial inhibition of UGPase activity is the trigger of compensated cell enlargement (CCE) in palisade tissue cells, this metabolic step is not critical for the formation of interlocking puzzle-shaped pavement cells in epidermal tissue. Finally, given that pavement cells were significantly smaller in the *fugu5* mutants, this indicates that CCE does not occur in the epidermis and that the reduced UI could be a result of restricted cell growth (**Figure 2G**).

### Potential Effect of Excess PPi on MT Dynamics

In plant leaves PPi is located predominantly in the cytosol at 0.2–0.3 mM (Weiner et al., 1987; Heinonen, 2001). We reported previously that the PPi level in *fugu5* was ~ 2.5-fold higher than in the WT (Ferjani et al., 2011), which is roughly estimated to be 0.5–0.75 mM. In this study, PPi at 1–10 mM reduced the tubulin polymerization rate in a concentration-dependent manner *in vitro* (**Figure S8**); these concentrations are higher than those reported *in vivo*. To address this discrepancy, we live imaged cortical MTs in the *fugu5* background. Based on the UI values, we expected to find clear differences in the arrangement of cortical MTs and/or their dynamics. However, the relative MT length and density were unexpectedly comparable between *fugu5* and the WT. By contrast, following oryzalin treatment at low concentrations, *fugu5* cortical MTs were slightly fragmented (reduced relative MT length) and sparse (reduced relative MT density), while the WT MTs were intact. Therefore, the increase in the PPi level in *fugu5* did not affect the arrangement but interfered with MT dynamics *in vivo*. This was supported by the fact that reduced MT length, density, and growth rate (velocity) in *fugu5* were significantly enhanced in *an-1 fugu5*. Together, the above results suggest that FUGU5 promotes MT growth and that it genetically interacts with AN to influence MT dynamics. However, the mechanism underlying this interaction is unclear and should be the focus of future research.

As mentioned above, several proteins of the ROP family together with the plant hormone auxin have been proposed to play a key role in shaping pavement cells, in which MT dynamics are of central importance. So, what are the implications of the above observations on *fugu5* MTs within the cell? MT polymerization rates *in vivo* are approximately 5- to 10-fold higher than those measured using purified tubulin *in vitro* (Cassimeris, 1993). Provided that the excess PPi level in *fugu5* partially inhibits gluconeogenesis and *de novo* protein synthesis *in vivo* (Ferjani et al., 2011), MT-associated proteins or other factors could be targets of suppression by PPi, even at concentrations lower than those used in the *in vitro* assay. If true, this would provide a plausible explanation for the *in vivo versus in vitro* discrepancy, but more research on the effects of PPi on MT-associated proteins is needed to resolve this issue.

### Perturbation of Leaf Flatness Due to Mechanical Buckling Between Adjacent Pavement Cells

Striking phenotypic similarities exist between *an-1* and *fugu5-1* mutant cotyledons. In fact, both single mutants exhibited oblong cotyledons, yet this phenotypic trait is controlled by different mechanisms. *fugu5-1* cotyledons are oblong because the cell number along their medio-lateral axis does not increase during postembryonic development (Ferjani et al., 2011). By contrast, *an-1* cotyledons are oblong due to restricted polar cell expansion along their medio-lateral axis and are thick due to excessive elongation of mesophyll cells along the adaxial-abaxial axis (Kim et al., 2002; Minamisawa et al., 2011). Here, pavement cell phenotyping revealed that in addition to the above similarities at the organ level, these two mutants shared a decreased complexity of pavement cells. Henceforth, *an-1 fugu5-1* double mutants were analyzed to evaluate whether their genetic interaction influences pavement cells.

Interestingly, live imaging of MTs revealed that their dynamics in *an-1 fugu5-1* are more severely affected than their respective parental lines (**Figure 6**). The most striking phenotype of *an-1 fugu5-1* cotyledons was their wavy surface, enhanced thickness (**Figure 3**), and higher cell density per unit length (**Figure S11**). The wavy surface was restored upon PPi removal in *an-1 fugu5-1 AVP1_pro_::IPP1#*8-3, which had notably wider cotyledons (**Figure 5**). Therefore, alleviation of the restricted organ expansion along the medio-lateral axis is sufficient to re-establish the smooth organ surface. Consistent with this, in our computational model, restriction of organ widening alone reproduced the 3D-protruding cells and the wavy surface characteristic of *an-1 fugu5-1*. The above interpretation highlights the loss of growth coordination along two major axes and the consequences on organ global morphology, yet it does not take into account the individual and/or local response of pavement cells. Hence, the twisting of pavement cells observed around stomata (**Figure 3J**) may reflect a mechanical conflict between guard cells and pavement cells, whereby MTs responsiveness to stress is different (Hervieux et al., 2017). Although single mutations in various genes reportedly disrupt leaf flattening, the combination of *an-1* and *fugu5-1* mutations indicated that this important trait can be altered by severely restricting cell proliferation and expansion along the leaf medio-lateral axis, adding to our knowledge of the genetic regulation of this important trait.

Taken together, our results provide valuable information on the effect of the accumulation of unhydrolyzed PPi on plant development *in vivo*. While PPi quantification and visualization are challenging (Gdula et al., 1998; Heinonen, 2001; and references therein), excess PPi resulted in visible phenotypes, such as compensation in the palisade tissue and defects in epidermal cell differentiation. Henceforth, we will focus on verifying how PPi exerts differential effects on different cell types, and whether the PPi concentration varies among plant tissues and cell types. Ultimately, we aim to identify key cellular components and/or critical metabolic steps, an important topic considering the universality of PPi homeostasis in all kingdoms of life.

## Data Availability Statement

The raw data supporting the conclusions of this article will be made available by the authors, without undue reservation, to any qualified researcher.

## Author Contributions

SG performed the experiments, analyzed the data, and wrote the article. YO performed live imaging of MT dynamics, and analyzed the data. HT-I performed computational modeling of the mechanical buckling, and analyzed the data. HT directed and funded the study. AF conceived the project, designed and funded the study, and wrote the paper with input from all co-authors. All authors read and approved the final manuscript.

## Funding

This work was supported by the Ministry of Education, Culture, Sports, Science and Technology of Japan [Grant-in-Aid for Encouragement of Young Scientists (B) (21770036 to AF); Grant-in-Aid for Scientific Research (B) (16H04803 to AF; 18H02469 to YO); Grant-in-Aid for Scientific Research on Innovative Areas (25113002 to AF and HT; 16H06280 and 19H05677 to YO); Grant-in-Aid for Scientific Research on Innovative Areas (18H05487 to AF); Grant-in-Aid for Scientific Research on Innovative Areas (19H05672 to HT); Grant-in-Aid for Scientific Research (C) (26520207 to HT-I)]; BIO-NEXT project from Okazaki Institute for Integrative Bioscience (KK and HT); and The Naito Foundation.

## Conflict of Interest

The authors declare that the research was conducted in the absence of any commercial or financial relationships that could be construed as a potential conflict of interest.
