# Supplementary material for: Excess Pyrophosphate Restrains Pavement Cell Morphogenesis and Alters Organ Flatness in Arabidopsis thaliana
Source: Front Plant Sci. 2020 Feb 21;11:31. doi: 10.3389/fpls.2020.00031 (PMC7047283; doi:10.3389/fpls.2020.00031)
Supplement: Supplementary file 1 [file Presentation_1.pptx]

## Slide 1
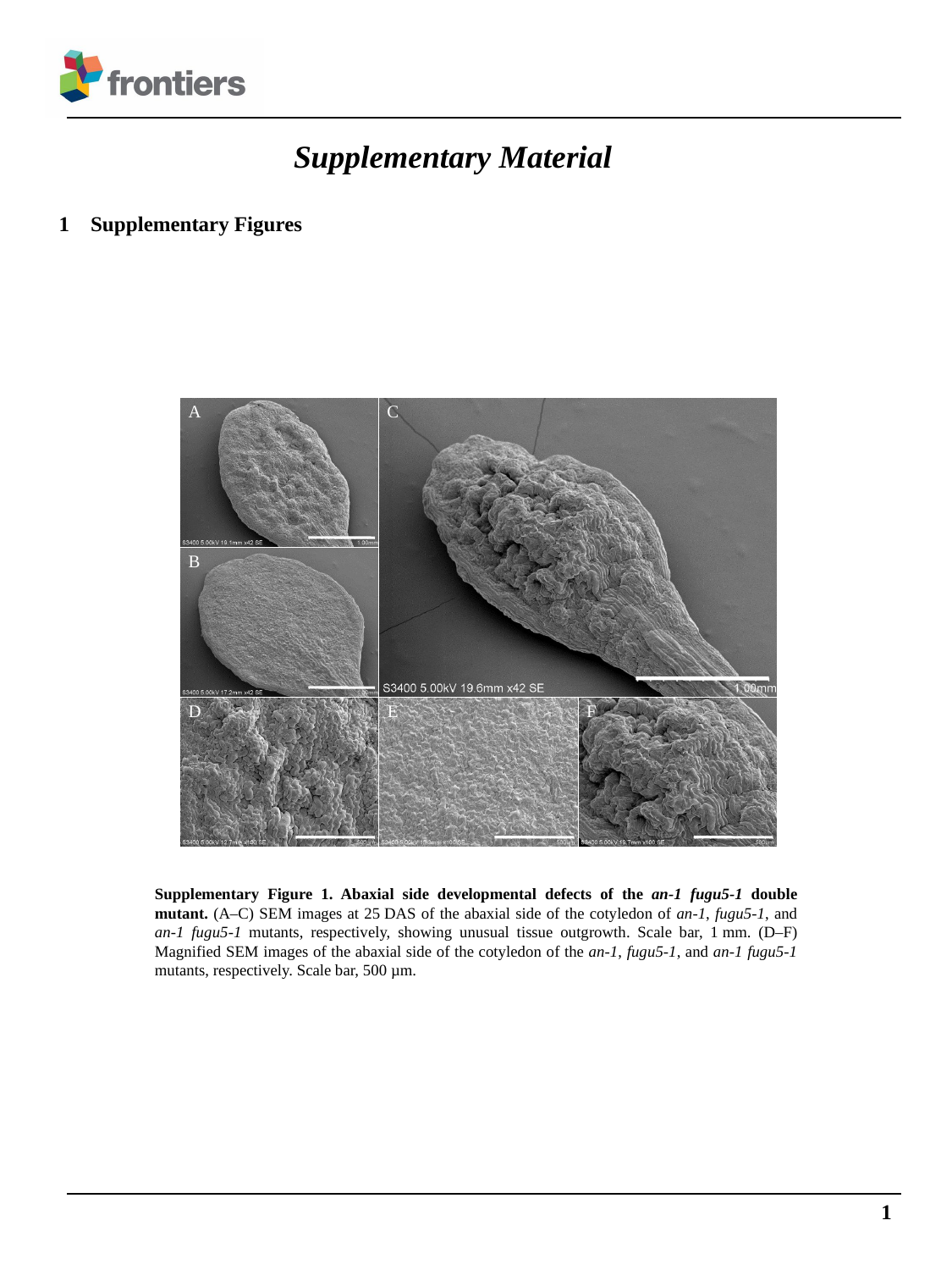

Supplementary Material
1 Supplementary Figures
A
C
B
D
E
F
Supplementary Figure 1. Abaxial side developmental defects of the an-1 fugu5-1 double mutant. (A–C) SEM images at 25 DAS of the abaxial side of the cotyledon of an-1, fugu5-1, and an-1 fugu5-1 mutants, respectively, showing unusual tissue outgrowth. Scale bar, 1 mm. (D–F) Magnified SEM images of the abaxial side of the cotyledon of the an-1, fugu5-1, and an-1 fugu5-1 mutants, respectively. Scale bar, 500 µm.
1

## Slide 2
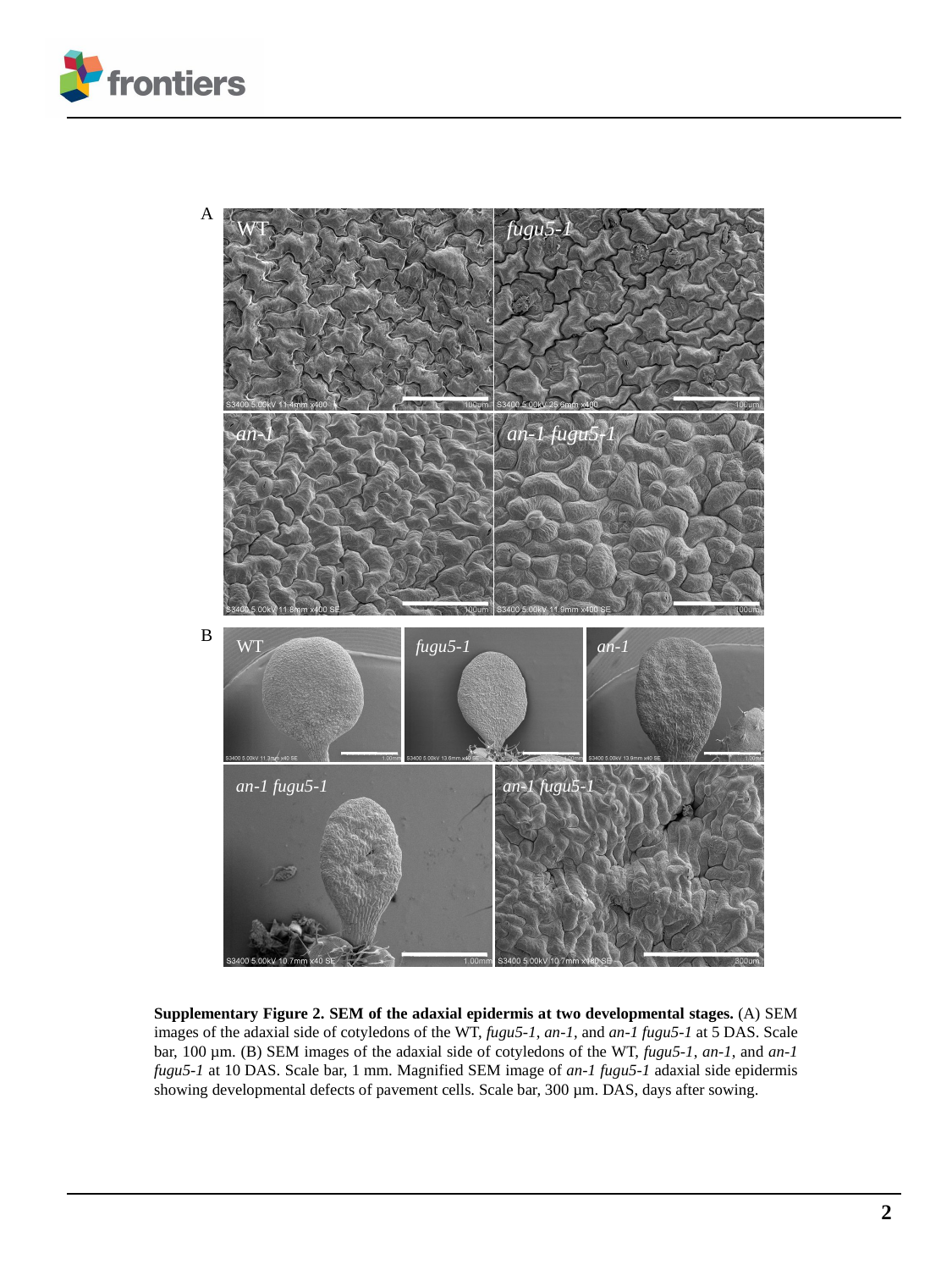

A
WT
fugu5-1
an-1
an-1 fugu5-1
WT
fugu5-1
an-1
an-1 fugu5-1
an-1 fugu5-1
B
Supplementary Figure 2. SEM of the adaxial epidermis at two developmental stages. (A) SEM images of the adaxial side of cotyledons of the WT, fugu5-1, an-1, and an-1 fugu5-1 at 5 DAS. Scale bar, 100 µm. (B) SEM images of the adaxial side of cotyledons of the WT, fugu5-1, an-1, and an-1 fugu5-1 at 10 DAS. Scale bar, 1 mm. Magnified SEM image of an-1 fugu5-1 adaxial side epidermis showing developmental defects of pavement cells. Scale bar, 300 µm. DAS, days after sowing.
2

## Slide 3
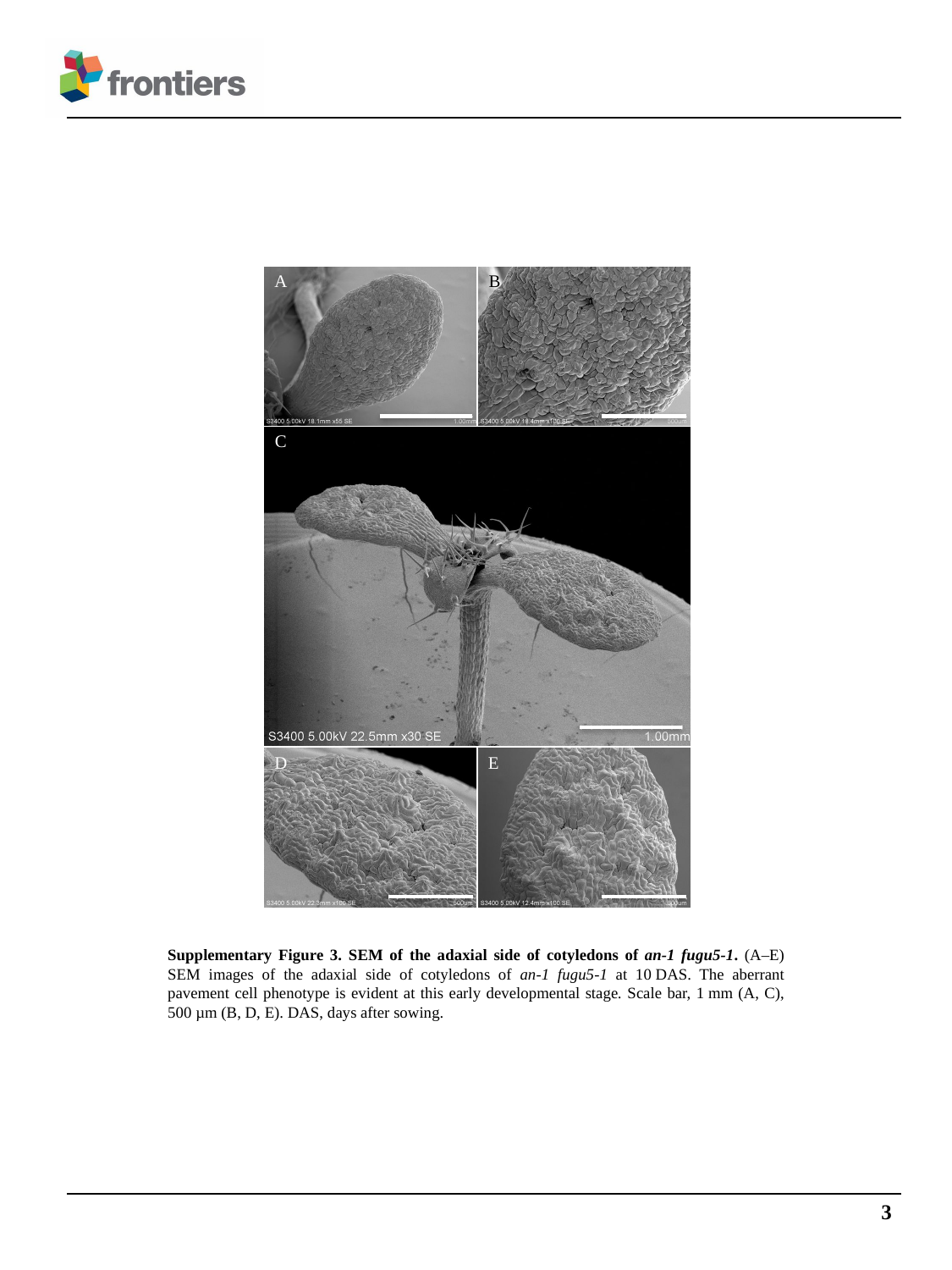

A
B
C
D
E
Supplementary Figure 3. SEM of the adaxial side of cotyledons of an-1 fugu5-1. (A–E) SEM images of the adaxial side of cotyledons of an-1 fugu5-1 at 10 DAS. The aberrant pavement cell phenotype is evident at this early developmental stage. Scale bar, 1 mm (A, C), 500 µm (B, D, E). DAS, days after sowing.
3

## Slide 4
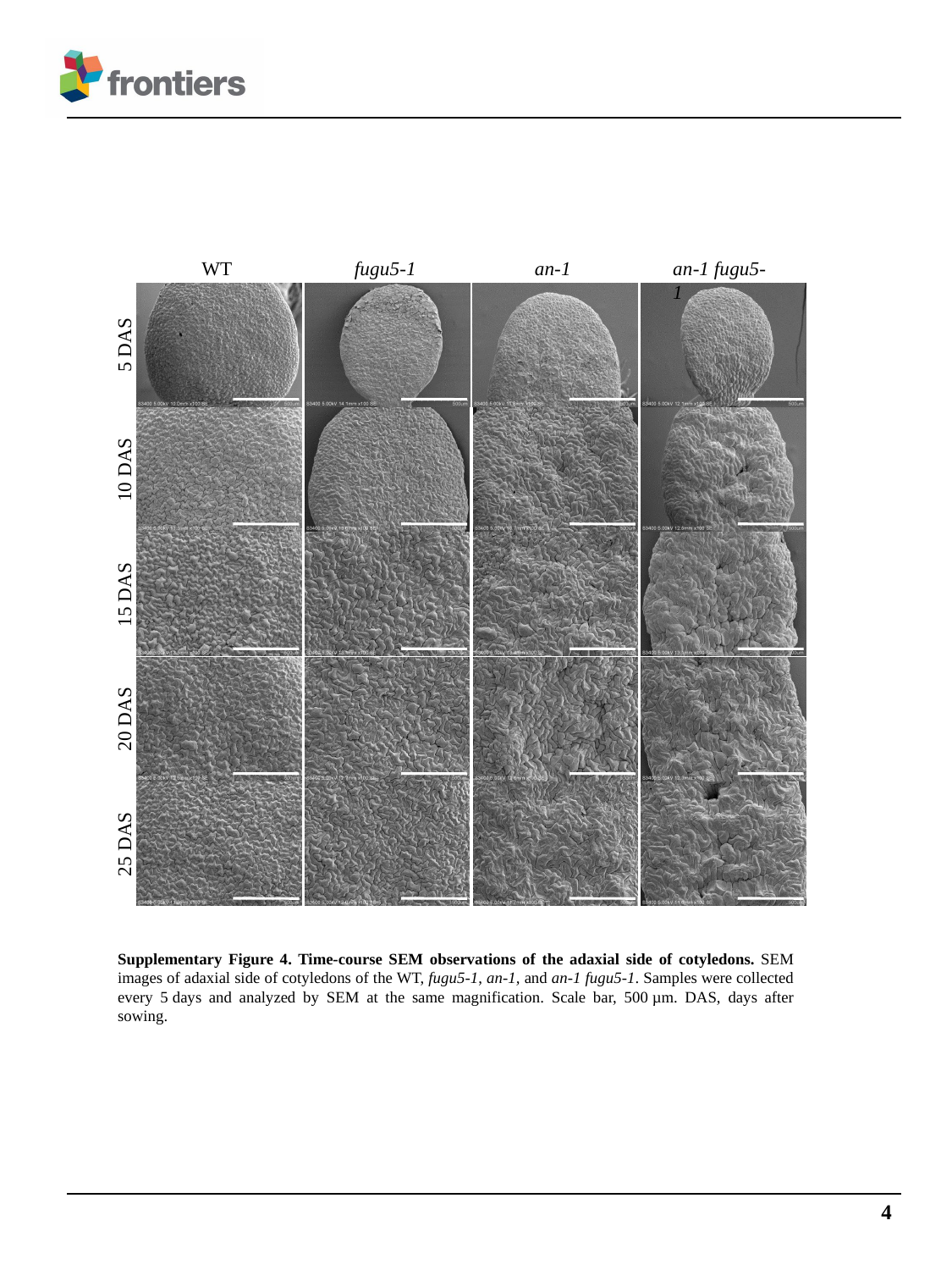

WT
fugu5-1
an-1
an-1 fugu5-1
5 DAS
10 DAS
15 DAS
20 DAS
25 DAS
Supplementary Figure 4. Time-course SEM observations of the adaxial side of cotyledons. SEM images of adaxial side of cotyledons of the WT, fugu5-1, an-1, and an-1 fugu5-1. Samples were collected every 5 days and analyzed by SEM at the same magnification. Scale bar, 500 µm. DAS, days after sowing.
4

## Slide 5
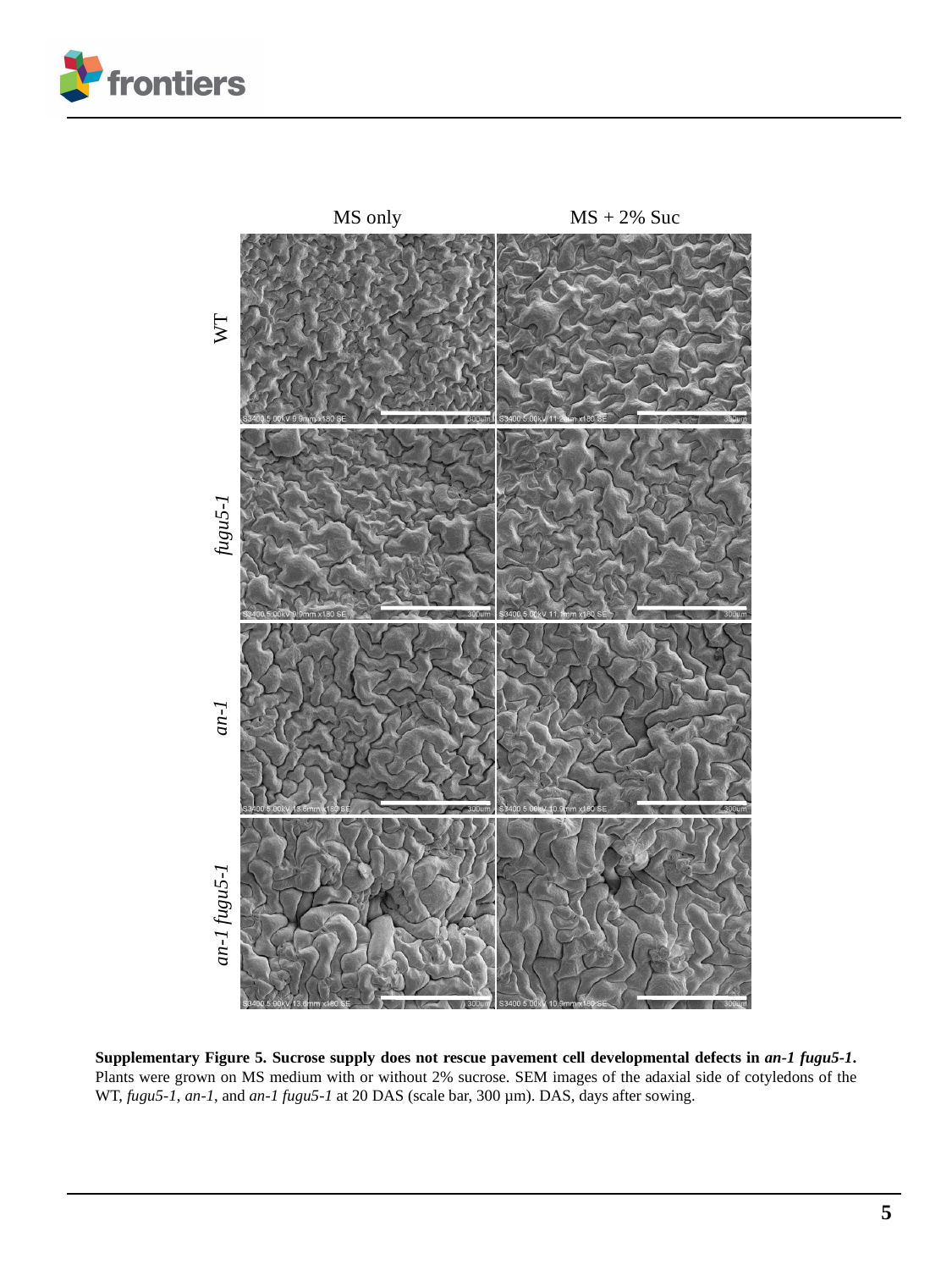

MS only
MS + 2% Suc
WT
fugu5-1
an-1
an-1 fugu5-1
Supplementary Figure 5. Sucrose supply does not rescue pavement cell developmental defects in an-1 fugu5-1. Plants were grown on MS medium with or without 2% sucrose. SEM images of the adaxial side of cotyledons of the WT, fugu5-1, an-1, and an-1 fugu5-1 at 20 DAS (scale bar, 300 µm). DAS, days after sowing.
5

## Slide 6
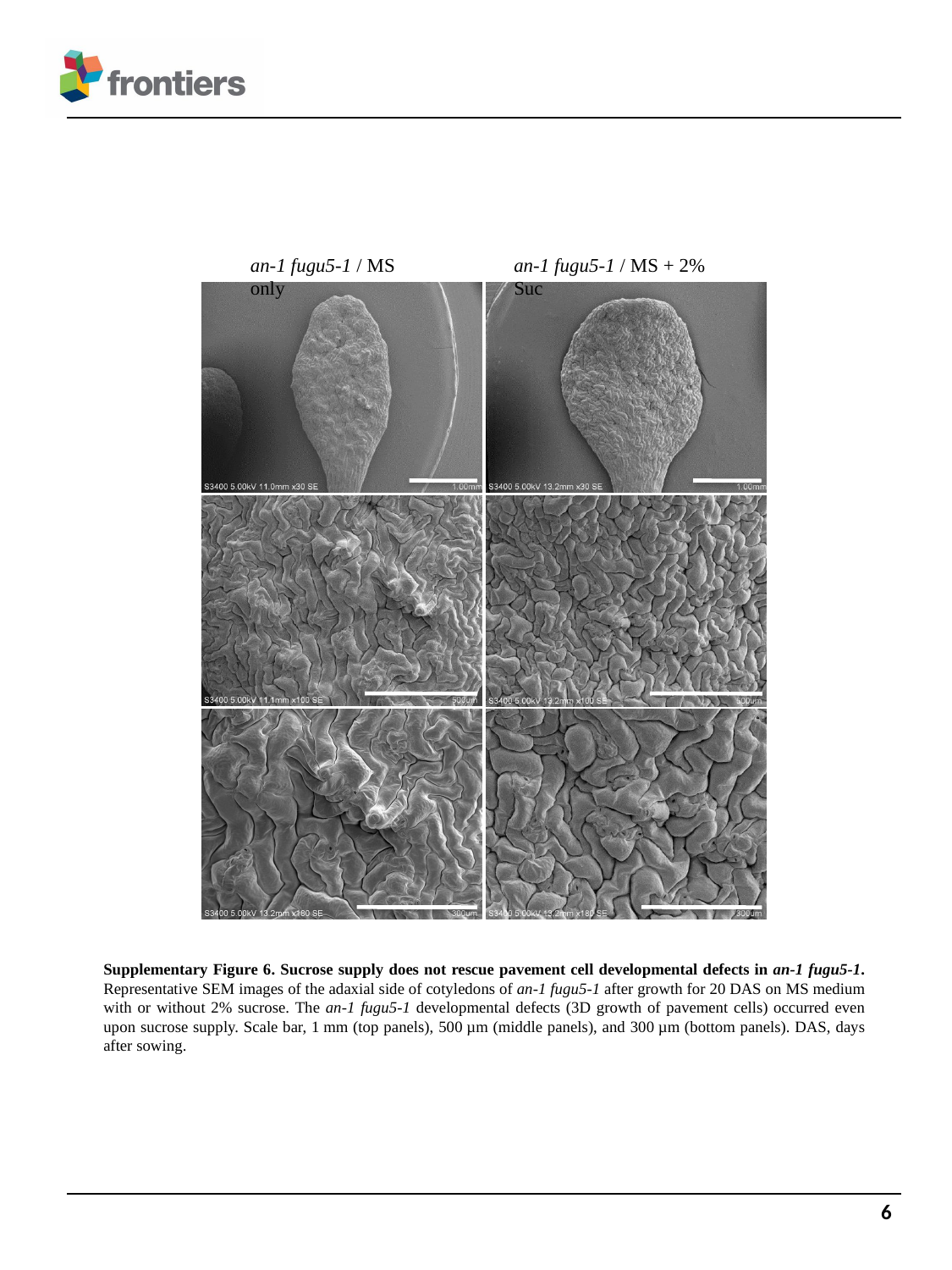

an-1 fugu5-1 / MS only
an-1 fugu5-1 / MS + 2% Suc
Supplementary Figure 6. Sucrose supply does not rescue pavement cell developmental defects in an-1 fugu5-1. Representative SEM images of the adaxial side of cotyledons of an-1 fugu5-1 after growth for 20 DAS on MS medium with or without 2% sucrose. The an-1 fugu5-1 developmental defects (3D growth of pavement cells) occurred even upon sucrose supply. Scale bar, 1 mm (top panels), 500 µm (middle panels), and 300 µm (bottom panels). DAS, days after sowing.
6

## Slide 7
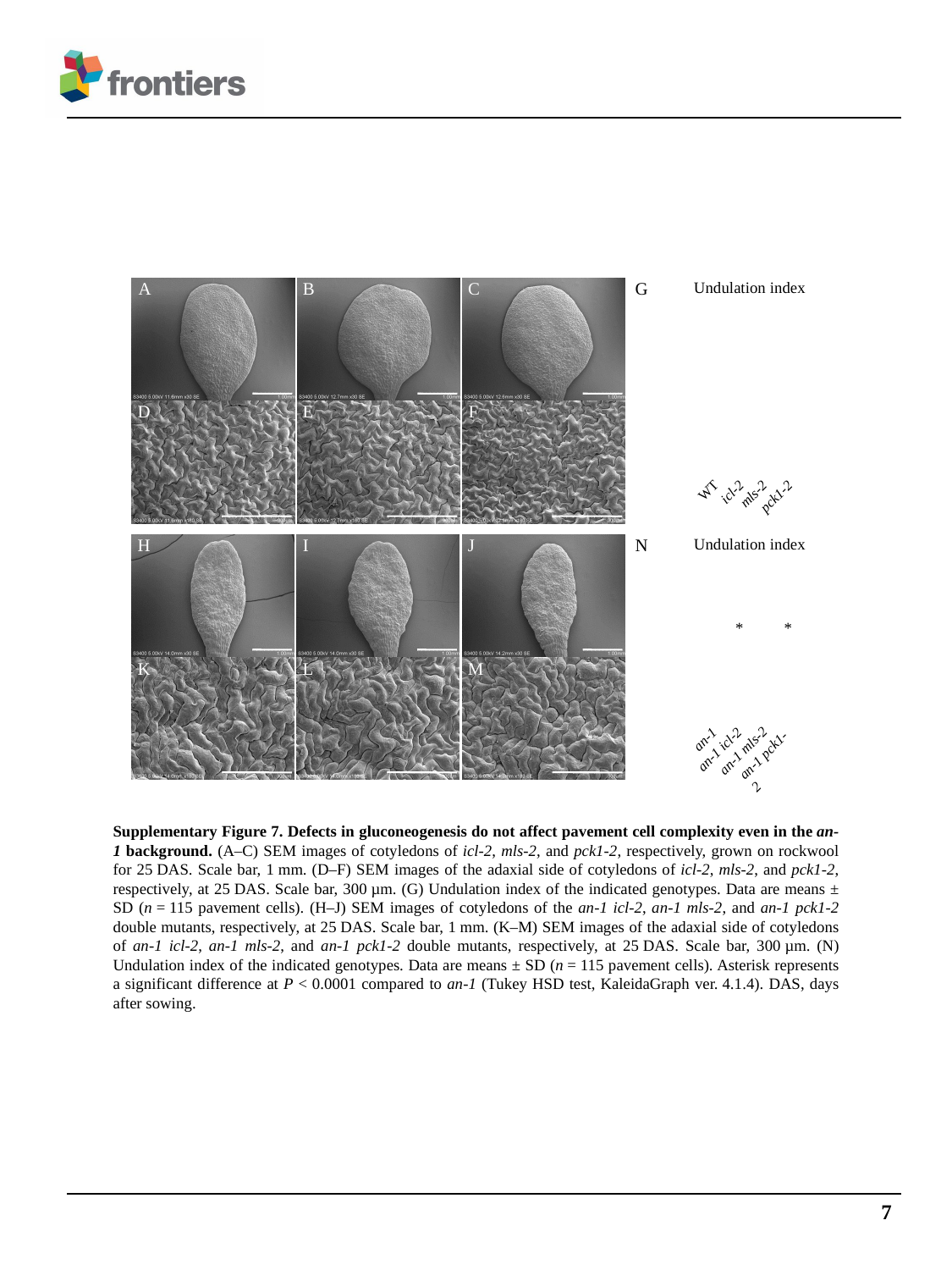

A
B
C
D
E
F
G
Undulation index
WT
icl-2
mls-2
pck1-2
H
I
J
K
L
M
N
Undulation index
*
*
an-1
an-1 icl-2
an-1 mls-2
an-1 pck1-2
Supplementary Figure 7. Defects in gluconeogenesis do not affect pavement cell complexity even in the an-1 background. (A–C) SEM images of cotyledons of icl-2, mls-2, and pck1-2, respectively, grown on rockwool for 25 DAS. Scale bar, 1 mm. (D–F) SEM images of the adaxial side of cotyledons of icl-2, mls-2, and pck1-2, respectively, at 25 DAS. Scale bar, 300 µm. (G) Undulation index of the indicated genotypes. Data are means ± SD (n = 115 pavement cells). (H–J) SEM images of cotyledons of the an-1 icl-2, an-1 mls-2, and an-1 pck1-2 double mutants, respectively, at 25 DAS. Scale bar, 1 mm. (K–M) SEM images of the adaxial side of cotyledons of an-1 icl-2, an-1 mls-2, and an-1 pck1-2 double mutants, respectively, at 25 DAS. Scale bar, 300 µm. (N) Undulation index of the indicated genotypes. Data are means ± SD (n = 115 pavement cells). Asterisk represents a significant difference at P < 0.0001 compared to an-1 (Tukey HSD test, KaleidaGraph ver. 4.1.4). DAS, days after sowing.
7

## Slide 8
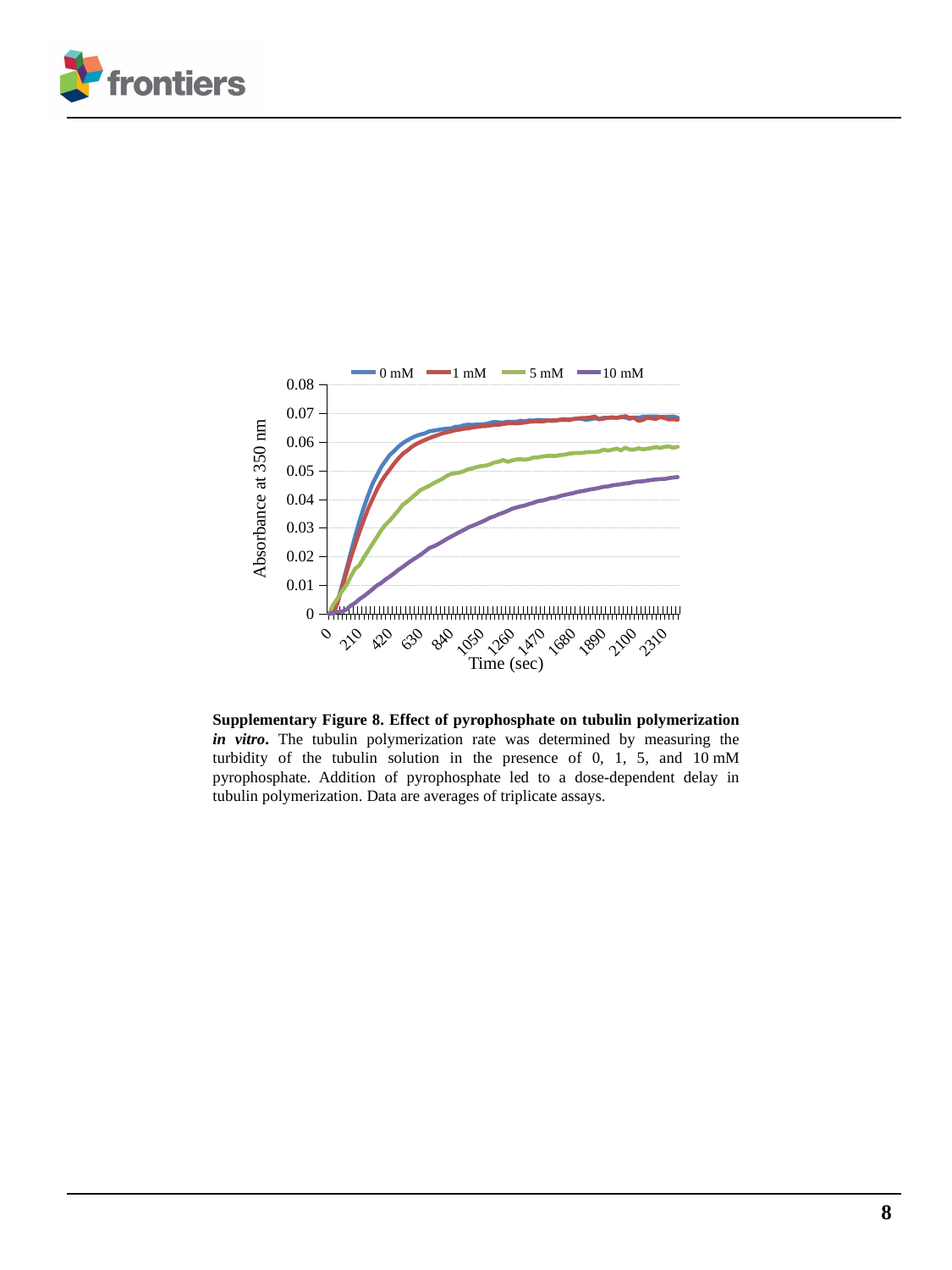

### Chart
| Category | 0 mM | 1 mM | 5 mM | 10 mM |
|---|---|---|---|---|
| 0 | 0.0 | 0.0 | 0.0 | 0.0 |
| 30 | -0.0004333333333333342 | 0.0003333333333333313 | 0.003166666666666665 | 0.0005333333333333324 |
| 60 | 0.004466666666666667 | 0.003933333333333333 | 0.005333333333333331 | 0.0006666666666666673 |
| 90 | 0.009766666666666667 | 0.009166666666666668 | 0.007933333333333334 | 0.000833333333333334 |
| 120 | 0.015433333333333334 | 0.014233333333333332 | 0.009999999999999997 | 0.0015000000000000013 |
| 150 | 0.02123333333333333 | 0.019366666666666667 | 0.012933333333333333 | 0.0028666666666666654 |
| 180 | 0.027 | 0.024066666666666667 | 0.01576666666666666 | 0.0038 |
| 210 | 0.03233333333333333 | 0.028499999999999998 | 0.016966666666666665 | 0.005133333333333334 |
| 240 | 0.037099999999999994 | 0.0327 | 0.0196 | 0.006133333333333335 |
| 270 | 0.0414 | 0.036666666666666674 | 0.021999999999999992 | 0.007333333333333333 |
| 300 | 0.045399999999999996 | 0.04006666666666667 | 0.024433333333333335 | 0.008600000000000003 |
| 330 | 0.04836666666666667 | 0.043366666666666664 | 0.02676666666666666 | 0.009900000000000004 |
| 360 | 0.051266666666666676 | 0.0462 | 0.029166666666666664 | 0.010766666666666667 |
| 390 | 0.05346666666666666 | 0.04843333333333333 | 0.031166666666666665 | 0.012033333333333333 |
| 420 | 0.05556666666666666 | 0.050499999999999996 | 0.03263333333333334 | 0.01306666666666667 |
| 450 | 0.0569 | 0.05256666666666667 | 0.03443333333333334 | 0.014199999999999997 |
| 480 | 0.05846666666666667 | 0.054366666666666674 | 0.03623333333333333 | 0.015433333333333334 |
| 510 | 0.05966666666666665 | 0.055966666666666665 | 0.038200000000000005 | 0.016433333333333335 |
| 540 | 0.06059999999999999 | 0.057066666666666675 | 0.039233333333333335 | 0.017599999999999998 |
| 570 | 0.06143333333333333 | 0.0583 | 0.040600000000000004 | 0.018666666666666668 |
| 600 | 0.062133333333333325 | 0.05926666666666667 | 0.04189999999999999 | 0.019600000000000003 |
| 630 | 0.06263333333333333 | 0.06 | 0.04323333333333334 | 0.0206 |
| 660 | 0.06303333333333333 | 0.0607 | 0.04393333333333333 | 0.021733333333333337 |
| 690 | 0.06369999999999999 | 0.06133333333333333 | 0.0447 | 0.022966666666666673 |
| 720 | 0.06396666666666666 | 0.06193333333333333 | 0.0456 | 0.023533333333333333 |
| 750 | 0.06423333333333334 | 0.062433333333333334 | 0.04636666666666667 | 0.0243 |
| 780 | 0.06449999999999999 | 0.06303333333333333 | 0.04713333333333333 | 0.0252 |
| 810 | 0.06466666666666666 | 0.06336666666666667 | 0.048133333333333334 | 0.0261 |
| 840 | 0.0647 | 0.06369999999999999 | 0.04886666666666666 | 0.026933333333333337 |
| 870 | 0.06533333333333333 | 0.06413333333333333 | 0.0491 | 0.027733333333333332 |
| 900 | 0.0654 | 0.06433333333333334 | 0.04936666666666666 | 0.028600000000000004 |
| 930 | 0.06586666666666667 | 0.06466666666666666 | 0.04986666666666667 | 0.029333333333333333 |
| 960 | 0.06606666666666666 | 0.06473333333333334 | 0.050533333333333326 | 0.030200000000000005 |
| 990 | 0.066 | 0.06513333333333333 | 0.050800000000000005 | 0.030800000000000004 |
| 1020 | 0.06613333333333334 | 0.0653 | 0.051300000000000005 | 0.031433333333333334 |
| 1050 | 0.06616666666666667 | 0.06549999999999999 | 0.05163333333333333 | 0.0321 |
| 1080 | 0.0663 | 0.0656 | 0.05176666666666666 | 0.0328 |
| 1110 | 0.0667 | 0.06576666666666667 | 0.05223333333333333 | 0.033600000000000005 |
| 1140 | 0.06706666666666666 | 0.06603333333333333 | 0.052899999999999996 | 0.0341 |
| 1170 | 0.06686666666666667 | 0.06603333333333332 | 0.05313333333333333 | 0.03479999999999999 |
| 1200 | 0.0668 | 0.0663 | 0.053733333333333334 | 0.035333333333333335 |
| 1230 | 0.0671 | 0.0665 | 0.0531 | 0.03596666666666666 |
| 1260 | 0.06703333333333332 | 0.06656666666666666 | 0.05356666666666666 | 0.036699999999999997 |
| 1290 | 0.06706666666666668 | 0.0665 | 0.053899999999999997 | 0.03713333333333333 |
| 1320 | 0.06743333333333333 | 0.06663333333333334 | 0.05399999999999999 | 0.037533333333333335 |
| 1350 | 0.06726666666666666 | 0.06683333333333334 | 0.05383333333333332 | 0.037866666666666667 |
| 1380 | 0.06756666666666666 | 0.06706666666666666 | 0.0541 | 0.0384 |
| 1410 | 0.06746666666666666 | 0.06716666666666667 | 0.0546 | 0.0388 |
| 1440 | 0.06773333333333333 | 0.06716666666666667 | 0.05463333333333333 | 0.039400000000000004 |
| 1470 | 0.06763333333333332 | 0.06716666666666667 | 0.054933333333333334 | 0.039566666666666674 |
| 1500 | 0.06763333333333334 | 0.06743333333333333 | 0.05513333333333333 | 0.04000000000000001 |
| 1530 | 0.06743333333333333 | 0.06753333333333333 | 0.05516666666666667 | 0.04046666666666667 |
| 1560 | 0.06743333333333333 | 0.06763333333333334 | 0.05513333333333333 | 0.040600000000000004 |
| 1590 | 0.06779999999999999 | 0.0677 | 0.055433333333333334 | 0.041133333333333334 |
| 1620 | 0.06799999999999999 | 0.0677 | 0.05556666666666666 | 0.04150000000000001 |
| 1650 | 0.0676 | 0.0679 | 0.05593333333333333 | 0.041833333333333333 |
| 1680 | 0.06799999999999999 | 0.068 | 0.056100000000000004 | 0.042133333333333335 |
| 1710 | 0.06816666666666665 | 0.0682 | 0.0562 | 0.04256666666666666 |
| 1740 | 0.0681 | 0.06836666666666667 | 0.05616666666666667 | 0.042833333333333334 |
| 1770 | 0.06773333333333333 | 0.0684 | 0.056433333333333335 | 0.043133333333333336 |
| 1800 | 0.06796666666666666 | 0.0686 | 0.056499999999999995 | 0.043466666666666674 |
| 1830 | 0.06826666666666666 | 0.06886666666666667 | 0.056499999999999995 | 0.043666666666666666 |
| 1860 | 0.06809999999999998 | 0.0679 | 0.0567 | 0.044000000000000004 |
| 1890 | 0.06843333333333333 | 0.06816666666666667 | 0.05733333333333334 | 0.04436666666666667 |
| 1920 | 0.0684 | 0.06846666666666666 | 0.057 | 0.044500000000000005 |
| 1950 | 0.06863333333333332 | 0.06856666666666666 | 0.05733333333333334 | 0.044866666666666666 |
| 1980 | 0.0684 | 0.06843333333333333 | 0.0577 | 0.04506666666666667 |
| 2010 | 0.06873333333333333 | 0.06866666666666667 | 0.057100000000000005 | 0.04526666666666667 |
| 2040 | 0.06856666666666665 | 0.06903333333333334 | 0.058033333333333326 | 0.045500000000000006 |
| 2070 | 0.0681 | 0.0684 | 0.05736666666666667 | 0.0457 |
| 2100 | 0.06856666666666665 | 0.0685 | 0.057333333333333326 | 0.046033333333333336 |
| 2130 | 0.06849999999999999 | 0.0674 | 0.05783333333333333 | 0.0462 |
| 2160 | 0.0688 | 0.0677 | 0.057466666666666666 | 0.04630000000000001 |
| 2190 | 0.06893333333333333 | 0.06846666666666666 | 0.057633333333333335 | 0.046533333333333336 |
| 2220 | 0.0689 | 0.06826666666666666 | 0.0579 | 0.04676666666666666 |
| 2250 | 0.06893333333333333 | 0.06803333333333333 | 0.05823333333333333 | 0.04693333333333335 |
| 2280 | 0.06873333333333333 | 0.0687 | 0.05793333333333334 | 0.047066666666666666 |
| 2310 | 0.06876666666666666 | 0.06836666666666667 | 0.05833333333333333 | 0.047099999999999996 |
| 2340 | 0.06879999999999999 | 0.06786666666666667 | 0.05846666666666667 | 0.0474 |
| 2370 | 0.0689 | 0.06796666666666666 | 0.057999999999999996 | 0.047599999999999996 |
| 2400 | 0.06853333333333333 | 0.06773333333333333 | 0.05833333333333334 | 0.04776666666666666 |0 mM
1 mM
5 mM
10 mM
Absorbance at 350 nm
Time (sec)
Supplementary Figure 8. Effect of pyrophosphate on tubulin polymerization in vitro. The tubulin polymerization rate was determined by measuring the turbidity of the tubulin solution in the presence of 0, 1, 5, and 10 mM pyrophosphate. Addition of pyrophosphate led to a dose-dependent delay in tubulin polymerization. Data are averages of triplicate assays.
8

## Slide 9
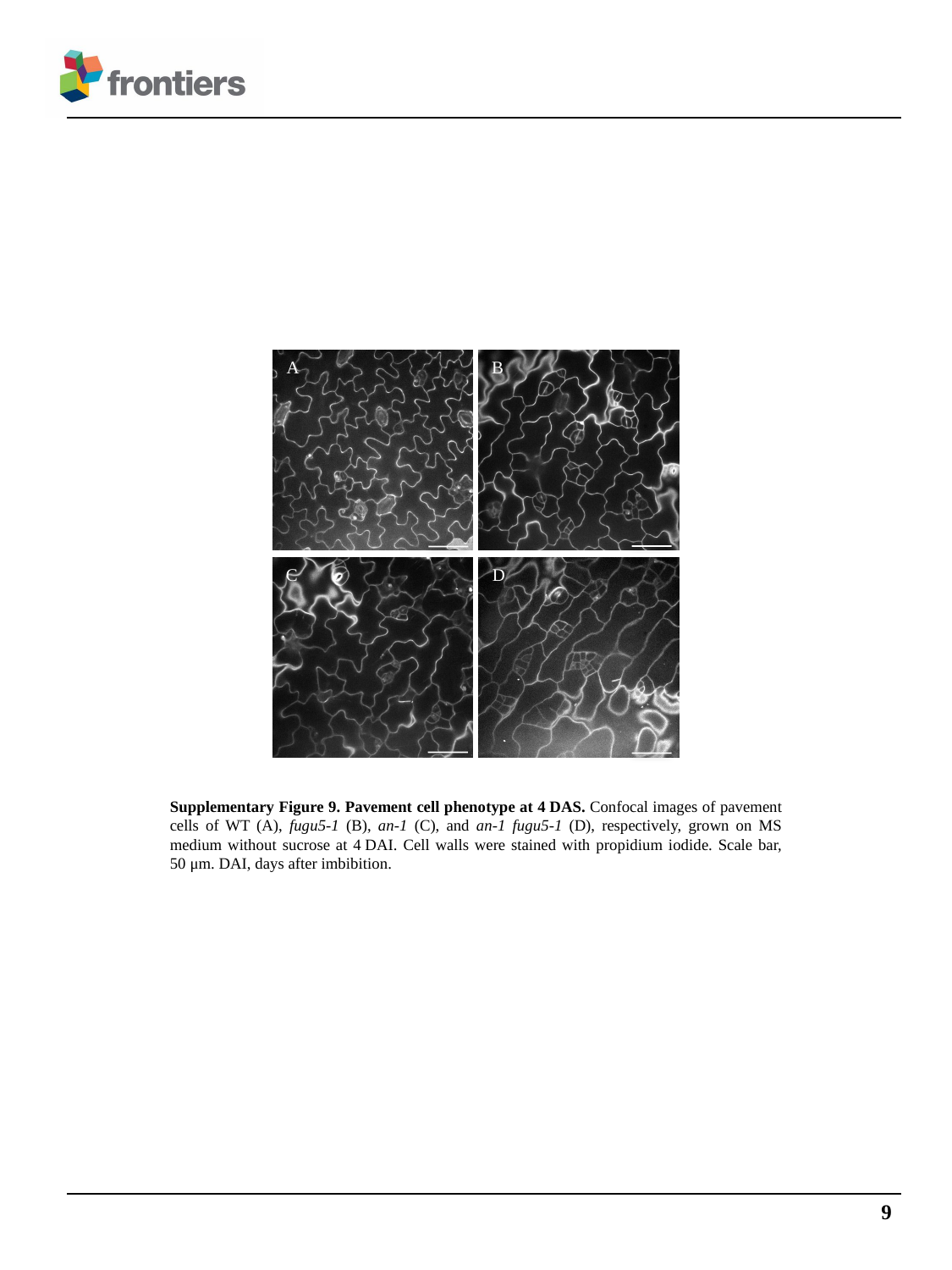

A
B
C
D
Supplementary Figure 9. Pavement cell phenotype at 4 DAS. Confocal images of pavement cells of WT (A), fugu5-1 (B), an-1 (C), and an-1 fugu5-1 (D), respectively, grown on MS medium without sucrose at 4 DAI. Cell walls were stained with propidium iodide. Scale bar, 50 μm. DAI, days after imbibition.
9

## Slide 10
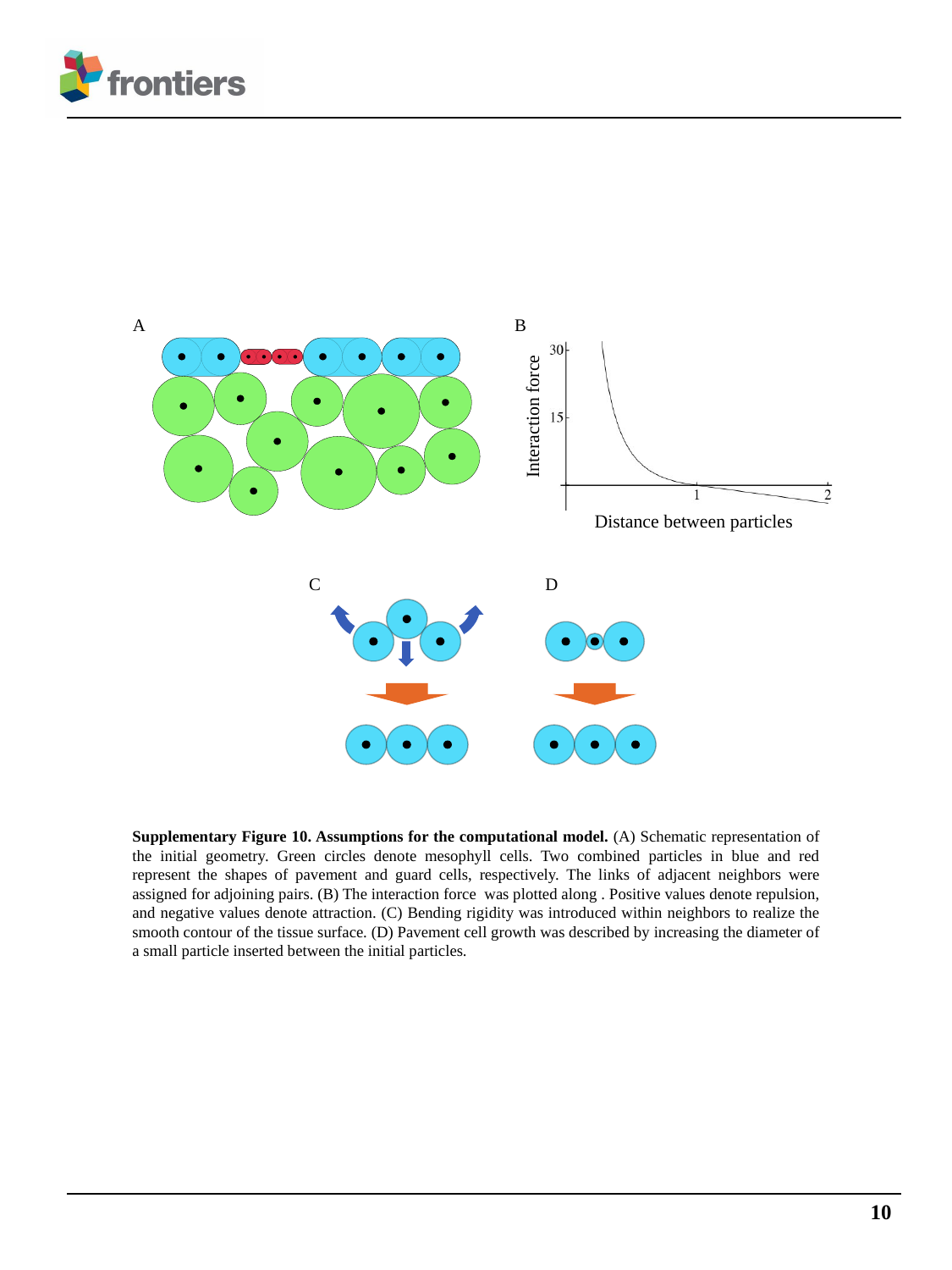

A
B
Interaction force
Distance between particles
C
D
10

## Slide 11
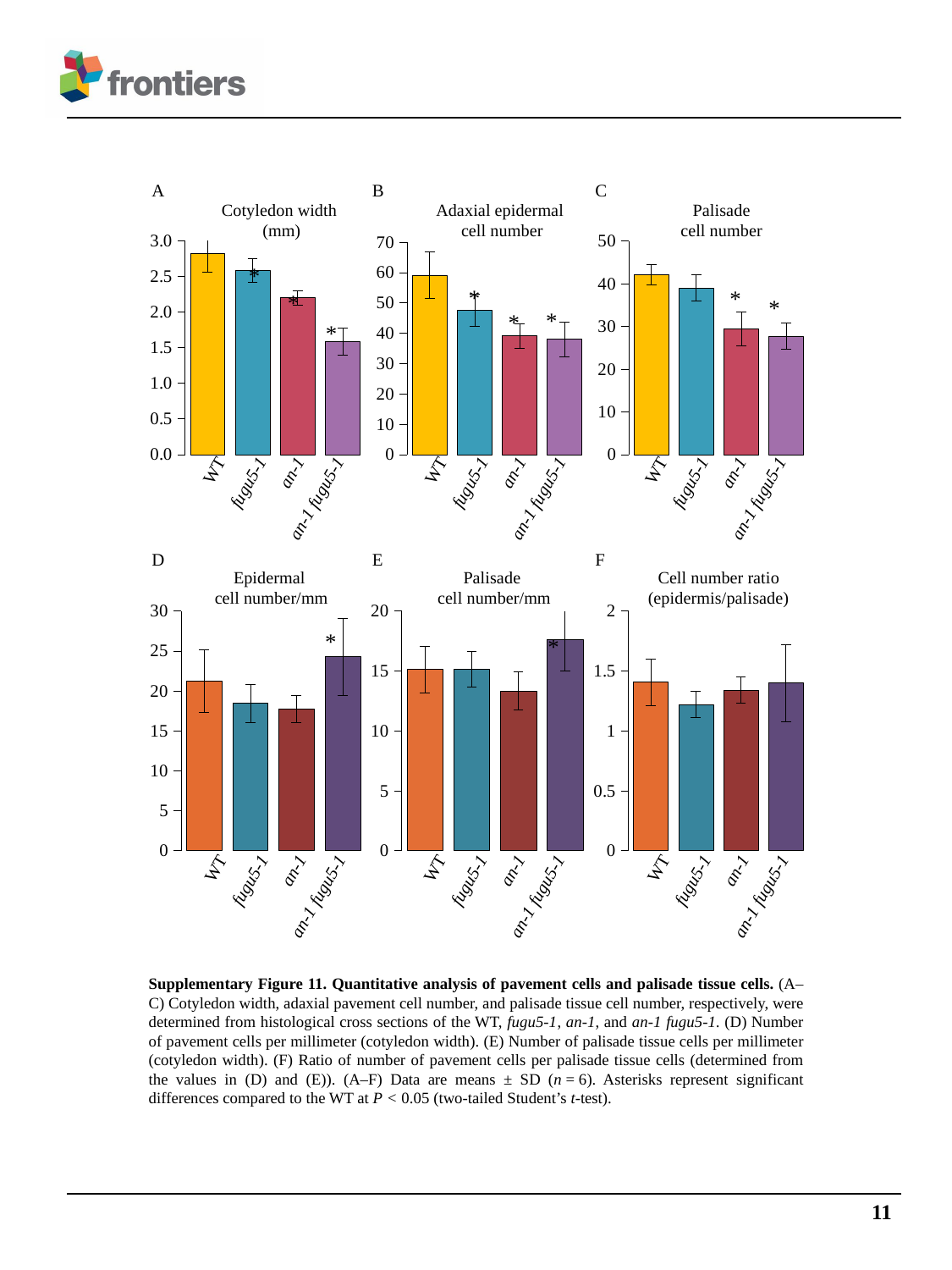

A
B
C
Cotyledon width
 (mm)
Adaxial epidermal
cell number
Palisade
cell number
### Chart
| Category | Length(mm) |
|---|---|
| WT | 2.815555555555555 |
| fugu5-1 | 2.583703703703704 |
| an-1 | 2.2 |
| an-1 fugu5-1 | 1.584444444444445 |
### Chart
| Category | Palisade |
|---|---|
| WT | 42.16666666666638 |
| fugu5-1 | 39.0 |
| an-1 | 29.375 |
| an-1 fugu5-1 | 27.66666666666667 |
### Chart
| Category | Epidermis |
|---|---|
| WT | 59.16666666666638 |
| fugu5-1 | 47.55555555555556 |
| an-1 | 39.125 |
| an-1 fugu5-1 | 38.0 |*
*
*
*
*
*
*
*
WT
an-1
fugu5-1
an-1 fugu5-1
WT
an-1
fugu5-1
an-1 fugu5-1
WT
an-1
fugu5-1
an-1 fugu5-1
D
E
F
Epidermal
 cell number/mm
Palisade
cell number/mm
Cell number ratio
(epidermis/palisade)
### Chart
| Category | Epedermis/Palisade |
|---|---|
| WT | 1.40657797198495 |
| fugu5-1 | 1.220178379863576 |
| an-1 | 1.340293421500717 |
| an-1 fugu5-1 | 1.398471303189045 |
### Chart
| Category | Epidermis/Length |
|---|---|
| WT | 21.2391967638236 |
| fugu5-1 | 18.46638244215507 |
| an-1 | 17.78083105319347 |
| an-1 fugu5-1 | 24.28918517654076 |
### Chart
| Category | Palisade/Length |
|---|---|
| WT | 15.12047699526451 |
| fugu5-1 | 15.14398486275591 |
| an-1 | 13.33923073872321 |
| an-1 fugu5-1 | 17.64730909868907 |*
*
WT
an-1
fugu5-1
an-1 fugu5-1
WT
an-1
fugu5-1
an-1 fugu5-1
WT
an-1
fugu5-1
an-1 fugu5-1
Supplementary Figure 11. Quantitative analysis of pavement cells and palisade tissue cells. (A–C) Cotyledon width, adaxial pavement cell number, and palisade tissue cell number, respectively, were determined from histological cross sections of the WT, fugu5-1, an-1, and an-1 fugu5-1. (D) Number of pavement cells per millimeter (cotyledon width). (E) Number of palisade tissue cells per millimeter (cotyledon width). (F) Ratio of number of pavement cells per palisade tissue cells (determined from the values in (D) and (E)). (A–F) Data are means ± SD (n = 6). Asterisks represent significant differences compared to the WT at P < 0.05 (two-tailed Student’s t-test).
11
